# Associations between sleep habits, quality, chronotype and depression in a large cross-sectional sample of Swedish adolescents

**DOI:** 10.1371/journal.pone.0293580

**Published:** 2023-11-02

**Authors:** Theresa Lemke, Sebastian Hökby, Danuta Wasserman, Vladimir Carli, Gergö Hadlaczky

**Affiliations:** 1 National Centre for Suicide Research and Prevention (NASP), Department of Learning, Informatics, Management and Ethics (LIME), Karolinska Institutet, Stockholm, Sweden; 2 National Centre for Suicide Research and Prevention (NASP), Centre for Health Economics, Informatics and Health Services Research (CHIS), Stockholm Health Care Services, Stockholm, Sweden; Sapienza University of Rome: Universita degli Studi di Roma La Sapienza, ITALY

## Abstract

**Objective:**

To investigate behavioral sleep habits, self-perceived quality of sleep, and chronotype, and to examine their association with clinically relevant levels of depression in Swedish adolescents.

**Method:**

Questionnaire data were obtained from a representative sample of Swedish adolescents (n = 8449; 50.8% girls; aged 12–16). Depression was defined as >13 BDI-II scores. Logistic regression modelling estimated the effects of sleep duration, sleep quality, and chronotype on depression, adjusted for socio-demographic factors.

**Results:**

On weekdays, approximately 46% of adolescents slept less than the recommended length of eight hours per night (depressed: 68%, non-depressed: 40%). On weekends, however, only 17% slept shorter than recommended. Short weekday sleep duration was more common among girls than boys (53% vs. 38%) and girls reported worse sleep quality. The regression model showed that depression was predicted by weekday sleep duration (OR = 0.773, p < .0001), sleep quality (OR = 0.327, p < .0001), and late chronotype (OR = 1.126, p = .0017), but not by weekend sleep duration. A 30-minute increase in weekday sleep duration was associated with about 10% lower odds of depression.

**Conclusions:**

A substantial proportion of Swedish adolescents do not seem to meet the sleep recommendations of eight hours per night. Short sleep duration on weekdays, poor sleep quality, and late chronotype were associated with increased risk of depression. Interventions promoting longer weekday sleep duration (e.g., later school start times) seem relevant in this context, but further research is needed to investigate the directionality and underlying mechanisms of these associations.

## Introduction

Sleep is an essential aspect of overall health and plays an important role for the mental and physical development of adolescents [1, 2]. Sleep problems are common during adolescence [3, 4] and are a serious public health concern, as inadequate sleep duration and poor sleep quality are associated with a range of adverse health and development outcomes such as reduced academic performance, emotion regulation, mood, well-being, and increased levels of risk-taking behavior and adiposity [5, 6].

### The human sleep-wake cycle

The human sleep-wake cycle is regulated by the circadian rhythm [7], which is an approximately 24-hour long cycle, driven internally by biological systems within the human body [8]. The circadian rhythm acts as an internal pacemaker for a range of physiologic functions and systems, including sleep, metabolism, the endocrine system, and the cardiovascular system [9]. Natural light exposure during the day and reduced light exposure (darkness) at night play a crucial role in synchronizing the internal circadian rhythm with the external 24-hour day-night cycle [7, 10]. Artificial light exposure also impacts this synchronization [11]. Light exposure regulates the production of the hormone melatonin, which is released with decreasing light and signals the transition from wakefulness to sleep [12]. The gold standard for determining an individuals’ circadian timing is therefore to measure the time in the evening at which endogenous melatonin levels rise above a certain threshold (Dim Light Melatonin Onset) [13].

The expression (phenotype) of how a person’s circadian rhythm is synchronized with the light-dark schedule, is referred to as the chronotype [14, 15]. In colloquial language, chronotype is what people commonly refer to when they say that they are a “morning person” or an “early bird” (i.e., early chronotype), which can be understood as the opposite of an “evening person” or a “night owl” (i.e., late chronotype). From a biological point of view, chronotype describes the biological tendency for a person’s timing of sleep and wakefulness in a 24-hour day [14, 15]. To accurately reflect this notion, the present study operationalizes chronotype using clock time as a continuous unit of measurement. For example, we may express an individual’s chronotype as being “02:45 a.m.”, or “03:00 a.m.”, which refers to the midpoint of these individuals’ sleep window, and where the former represents a 15-minutes earlier chronotype than the latter (see methods for a more detailed account).

### Sleep problems during adolescence

During adolescence, the circadian rhythm becomes delayed and the homeostatic sleep pressure (drive for sleep, which increases with longer periods of wakefulness) is reduced, which leads to a changed sleep-wake cycle, characterized by higher levels of alertness in the evening and later onset of sleep. This change happens until around the age of 20, after which adolescents’ sleep-wake cycle begins to shift back to earlier bedtimes [14]. In a contrary way, wake times on weekdays generally stay consistent throughout adolescence due to early mandated school start times, which ultimately increases the risk of shorter sleep duration among adolescents compared to younger children.

Apart from biological changes influencing adolescents’ sleep patterns, behavioral and social factors further contribute to a shift towards later bedtimes and a delay in sleep onset time. Examples include increased use of electronic media and artificial light exposure in the evening, energy drink consumption, and less parental involvement in setting bedtimes [16–20].

It is generally recommended that 13-18-year-olds sleep between 8–10 hours per night regularly, with advice against a sleep duration of fewer than 7 hours or more than 11 hours [21]. However, to occasionally sleep in on weekends to compensate for sleep debt is not necessarily unhealthy [22]. Nonetheless, short sleep duration has become an increasing problem in many parts of the world [4]. Prevalence estimates of sleep duration of fewer than 8 hours in adolescents range from 12% to 72% [23–25].

Apart from short sleep duration, many adolescents experience poor sleep quality [24]. Sleep quality is typically defined as a person’s subjective perception of and satisfaction with their sleep [26], but may also be influenced by actual sleep habits such as long sleep onset times, the number of awakenings during the night, and sleep disturbances such as nightmares [26, 27]. According to a systematic review, electronic and social media use is associated with adolescents experiencing restless sleep and night-time awakenings [28]. These findings are supported by adolescents’ perceptions of barriers to good sleep quality, which include stress, electronic media use, poor sleep habits, and rumination around bedtime [18, 29].

### The relationship between sleep and mental health

Adolescence is a developmental phase during which both sleep problems as well as mental health problems tend to have their onset [16, 30]. Worldwide, approximately 14% of 10-19-year-olds have a mental disorder [31] and suicide is the second leading cause of death among 15-19-year-olds [32]. One of the most common mental disorders in adolescents is depression, with prevalence estimates between 3–8% in Europe [33, 34] and 5–8% in Sweden [35].

Sleep problems appear to be a core symptom in many psychopathologies [36, 37]. For example, difficulty initiating or maintaining sleep and early awakenings with inability to return to sleep (symptoms of insomnia) and/or excessive sleep duration and daytime sleepiness (symptoms of hypersomnia) are central symptoms of mental disorders such as major depressive disorder and anxiety [38]. More specifically, sleep problems may also be causally implicated in comorbid psychopathologies [36].

A growing body of research suggests that the relationship between sleep problems and mental health problems may be bi-directional [39, 40]. However, sleep problems might be a stronger predictor for mental health problems than vice versa, according to a meta-analysis investigating the relationship between both objectively and subjectively measured sleep parameters and depression in adolescents [41].

### The relationship between depression and sleep problems in adolescents

A number of studies using substantially different sleep measures, such as polysomnography, sleep diaries or self-report questionnaires, have been carried out to investigate the relationship between sleep and mental health in adolescents. One meta-analysis that summarizes such studies found that shorter sleep duration is associated with an increased risk of mood problems and depressive disorders in adolescents [42]. There is some indication that not only short but also very long sleep duration is associated with depressive symptoms in adolescents [43]. Other sleep habits that characterize depressive disorders in among adolescents include later sleep onset times, longer sleep onset latency, and more frequent awakenings after sleep onset [41, 44].

There also seems to be a relationship between depression and adolescents’ subjectively perceived quality of sleep, such as feelings of not getting enough sleep and having difficulties getting up in the morning [44]. In fact, the association between severity of depression and sleep seems to be stronger for measures of perceived sleep quality, as opposed to measures of self-reported sleep behavior [45]. Since subjective sleep quality has been less studied than sleep habits in the context of depression [45], more studies investigating sleep quality and depression are warranted to understand this phenomenon.

Depression in adolescents may not just be associated with different parameters of the process of sleep itself, but also with chronotype (how a person’s circadian rhythm is synchronized with the 24-hour day/light cycle). It appears that a biological tendency for the sleep phase to be initiated at later times in the evening (a late chronotype) is associated with depression in both adult and adolescent populations [46]. A longitudinal study of adolescents found that late chronotype is associated with future risk of diagnosis of depression and increases in depressive symptoms, but the relationship seems bi-directional [47]. Several possible mechanisms may explain this relationship, including societal factors such as early school start times, that prevent late chronotypes from sleeping enough and thus increase their risk for adverse outcomes. Also, psychological factors (i.e., rumination in the evening), biological factors (i.e., gene variations associated with both circadian rhythm and mood), and light exposure (which may be reduced in depressed individuals and has an impact on the circadian rhythm) could explain the relationship between chronotype and depression [48].

### Study aims and goals

The primary aim of this study was to describe behavioral sleep habits and self-perceived quality of sleep in a large Swedish sample of adolescents aged 12 to 16 years. The secondary aim was to investigate the relationship between different sleep parameters and potentially clinically relevant levels of depression in this population. Such knowledge could inform the development of preventive interventions and clinical treatment for depression [49]. Perhaps more likely, improving the understanding of the relationship between sleep and depression could contribute to public health prevention efforts. For example, sleep habits could be targeted in mental health literacy programs and gatekeeper training (teachers, community leaders etc.), which can be valuable even to parents or teachers with overall limited health literacy. However, that possibility also depends upon knowledge of what characterizes abnormal sleeping behaviors in terms of clinical significance (i.e. cause for concern). Related to the first study aim, our goal is to use large-scale data to provide some normative sleep data for Swedish adolescents aged 12 to 16 years. Furthermore, our goal is to express the association between those sleep behaviors and depression in a binary fashion (i.e., low risk vs. high risk), to make findings transferrable to a wider audience, such as school boards, policy makers and gatekeepers.

## Methods

In this study, the Karolinska Sleep Questionnaire (KSQ), the Beck Depression Inventory-II (BDI-II) and socio-demographic data were used to evaluate sleep measures (habits, quality, and chronotype), depression, and socio-demographic variables.

### Study design and participants

This study analyzed cross-sectional baseline data from a representative sample of adolescents aged 12–16 years in Stockholm County, Sweden. The data was obtained from a digitalized questionnaire survey, where students responded to a health questionnaire while being in a classroom setting. The health questionnaires were administered to collect information from subjects during a cluster-randomized controlled trial evaluating a universal, school-based mental health promotion program in *n* = 116 elementary and junior high schools (baseline measurement conducted while students attended grade 7 or 8) in Stockholm County between 2016 and 2020 (ISRCTN trial registration number: 17583138) [50]. The content of the intervention “Youth Aware of Mental health” (YAM) has been described elsewhere [51]. Data were collected at baseline (*n* = 10299) and at 3-month and 12-month follow-up. Participant recruitment and baseline data collection took place between August 2016 and November 2018. The present study includes baseline data from all participants in the control and intervention group who were between 12 and 16 years old at the time of enrolment (*n* = 10288), excluding participants that were either younger or older (*n* = 11). For the main regression analysis of this study, *n* = 8449 participants (complete cases) were included (Table 1).

**Table 1:** Sample characteristics.

Variable	Total sample (*n* = 10288)		Main analysis sample (*n* = 8449)
	**N (%)**	**Missings, n (%)**	**N (%)**
**Gender**			
Boys	5179 (50.34)	89 (0.87)	4158 (49.21)
Girls	5020 (48.79)		4291 (50.79)
	**M (SD)**	**Missings, n (%)**	**M (SD)**
**Age** (12–16 years old)	13.98 (0.72)	31 (0.30)	13.98 (0.72)
**Socio-economic status** (range: 1–5)	4.47 (0.86)	57 (0.55)	4.51 (0.82)

*Notes*: M = Mean, SD = Standard deviation.

Total sample: Baseline data from all participants aged 12–16 years old.

Main analysis sample: Participants with complete data (included in regression analysis).

The main analysis sample (*n* = 8449) included 50.79% (*n* = 4291) girls and 49.21% (*n* = 4158) boys. The average age was 14 years (*SD* = 9 months). Self-perceived socioeconomic status can be interpreted as relatively high, as most participants reported that they often have enough money to do the same things as their friends (range 1–5, *M* = 4.51, *SD* = 0.82). Self-reported socioeconomic status was lower in girls (*M* = 4.48, *SD* = 0.84) compared to boys (*M* = 4.54, *SD* = 0.81; p = .0007, t = 3.391); however, the difference was small.

Written consent was obtained from the adolescents or from a legal guardian if the participant was under the age of 15. The questionnaires were pseudonymized but not entirely anonymous. During data collection, only the principal investigators had access to documents that could connect personal information with the pseudonymizing participant codes. De-identification followed a strict protocol and was only used to contact and provide help to participants who reported a high degree of suicidal planning or attempts within the past two weeks. Before participants provided personal information and contact information (name, personal/social security number, phone number), the data collectors informed them that they might be contacted in case of “serious health concerns”. All sensitive information was removed from the dataset after data collection completion and before the present data analyses began. The study was ethically approved by the regional ethics committee in Stockholm (diary number: 2016/2175-31/5) and was conducted in accordance with the Declaration of Helsinki.

### Measures

Questionnaire data were collected in a classroom setting using electronic, self-administered questionnaires. Sleep habits and sleep quality were measured using a modified, electronically adapted version of the Karolinska Sleep Questionnaire (KSQ) [52]. The KSQ is a self-assessment scale aiming to measure subjective nocturnal sleep and daytime sleepiness. The KSQ has been developed for an adult population and has been validated in a Swedish sample of adults. Factor analyses found that nocturnal sleep is comprised of three dimensions: sleep quality, non-restorative sleep, and sleep apnea. The dimensions sleep quality and non-restorative sleep have been shown to be moderately correlated with anxiety, depression, and stress [52].

#### Sleep habits

Self-reported sleep habits that were measured in the KSQ were bedtime, wake time, and time in bed before falling asleep (sleep onset latency) and were assessed separately for weekdays and weekends. Based on this information, sleep onset time, sleep duration, time in bed, and chronotype were calculated (see S1 Table for definitions and calculations). Regarding the reported bedtimes, a minority of responses ranged between 7:00 and 13:00 (weekdays: *n* = 1054; weekends: *n* = 715) and these were considered as data entry errors reflecting answers on a 12-hour scale instead of the actual 24-hour scale (thus, e.g., a bedtime of 7:00 was corrected to 19:00). The bedtime and wake time variables appeared heavy-tailed (bedtime weekdays: *M* = 22:30, *SD* = 1:07, kurtosis: 6.368; bedtime weekends: *M* = 24:08, *SD* = 1:47, kurtosis: 4.526; wake time weekdays: *M* = 6:52, *SD* = 1:15, kurtosis: 113.198; wake time weekends: *M* = 9:58, *SD* = 1:47, kurtosis: 11.166). We therefore removed outliers to improve normal distribution approximations. The bedtime and wake time variables were trimmed by removing outlier values beyond three standard deviations (*SD*) from the arithmetical mean (outliers per variable: bedtime weekdays: *n* = 167, bedtime weekends: *n* = 79; wake time weekdays: *n* = 73; wake time weekends: *n* = 113) [53]. This was compared to a median-based trimming technique [54], which retained fewer cases than the SD approach while resulting in similar means and standard deviations. The SD-trimmed variables were subsequently used for the calculation of the sleep variables of main interest, such as sleep duration. However, the resulting variables were not trimmed again as suggested by Leys and colleagues [55].

#### Chronotype

The chronotype is a measure of how a person’s circadian rhythm synchronizes with the 24-hour day in terms of the timing of this person’s sleep phase within a day [14, 15]. In the current study, chronotype was operationalized as the midpoint of sleep on weekends, adjusted for sleep debt, using the approach suggested by Roenneberg and colleagues [14, 22]. The adjustment is necessary because the midpoint of sleep on weekends appears to be a more precise indicator of chronotype than midpoint on weekdays, because sleep behavior is less likely to be affected by social obligations (i.e., school) [14]. However, the midpoint of sleep on weekends may be affected by sleep dept, especially in individuals with a late chronotype, who build up more sleep debt during weekdays compared to early chronotypes, and tend to compensate for this by sleeping in on weekends [15]. To adjust for sleep-debt, Roenneberg and colleagues [14, 22] suggest a mathematical correction, by first calculating the weekly average sleep duration and then subtracting half of the sleep compensation (difference between weekday sleep duration and weekly average) from the midpoint of sleep (S1 Table) [22]. For example, a person with an average sleep onset at 24:00 on weekends and weekend sleep duration of 10 hours has a sleep mid-point of 05:00 a.m. If this person sleeps 7 hours on weekdays (resulting in a weekly average sleep duration of 7.9h and 2.1h of sleep compensation), then their corrected sleep midpoint on weekends will be at 03:55 a.m. (05:00 a.m.—(2.1h/2)). However, some individuals might have equal or even more social obligations on weekends than on weekdays, which would prevent them from compensating for sleep debt that has been accumulated on weekdays by sleeping longer on weekends. Therefore, according to the approach by Roenneberg and colleagues (2019), the sleep midpoint on weekends is not adjusted for sleep debt in cases where sleep duration is shorter or equally long on weekends compared to weekdays.

#### Sleep quality

The modified KSQ included 7 items on the perceived quality of sleep (translated from Swedish to English): 1) *Difficulties falling asleep*. 2) *Difficulties waking up*. 3) *Repeated awakenings with difficulties falling asleep again*. 4) *Nightmares*. 5) *Not well-rested on awakening*. 6) *Premature awakenings*. 7) *Disturbed/restless sleep*. Participants indicated the frequency of having experienced these problems during the past 3 months on a 1–6 Likert scale ranging from “never” to “always (5 or more times per week)”. Scores were reverse-coded, with higher scores indicating better sleep quality. A sleep quality index was calculated as the average score of 7 KSQ items (complete cases). A score < 3 (meaning sleep quality problems that occur either “always (5 times or more per week)” or “most of the time (3–4 times per week)”) can be interpreted to indicate clinically meaningful problems relating to sleep quality, as this cut-off reflects the diagnostic criteria for insomnia disorder in the Diagnostic and Statistical Manual of Mental Disorders (5^th^ ed.; DSM-V) [38], according to which problems with sleep quality need to be present at least three times per week and has been validated as a cut-off score for the KSQ in an adult population [52].

The internal consistency of the 7-item sleep quality index in the main analysis sample (*n* = 8449) was satisfactory, as indicated by Cronbach’s alpha values of α = 0.79 and the McDonald’s omega values of ωh = 0.65 and omega-total ωt = 0.85. Reliability coefficients for the 7-item sleep quality index and for indices excluding one of the items at a time are shown in the supporting information (S2 Table). The omega reliability analysis showed that communalities (indicating the variance explained by the retained factors) were lowest for the items “nightmares” (0.28) and “premature awakenings” (0.27), hence, a sleep quality index without these items was calculated for sensitivity analyses.

#### Depression

Presence of clinical symptoms of depression was assessed using the Swedish translation of the Beck Depression Inventory-II (BDI-II) [56], a validated 21-item questionnaire for measuring depressive symptoms [57]. Symptom severity is rated on a 4-point Likert scale ranging from 0 (*Not at all*) to 3 (*Severely*), resulting in a maximum total score of 63. As this study investigates the relationship between sleep and depression, the items “Changes in sleep patterns” and “Tiredness or fatigue” were excluded from the calculation of the BDI-II scores. In addition, the item “Loss of sexual interest” was excluded following the assessment of the psychometric properties of BDI-II items in the main analysis sample (*n* = 8449). The item contributed little to the internal consistency of the scale (including the item did not improve omega, see S2 Table) and had the lowest correlation with the BDI score composed of the remaining items (r = 0.17). Descriptive statistics showed that it also had the lowest mean (*M* = 0.19, *SD* = 0.620) and the highest number of missing values (*n* = 194) among all BDI items. This suggests that the item “Loss of sexual interest” might measure something else than depression in our sample. The item was removed from the BDI-II score to avoid that it systematically biases the prevalence estimation for depression in the study.

To maintain the original score-range from 0–63 and utilize the validated and commonly used clinical cut-off of BDI > 13 for depression, values for the three excluded items were imputed using the mean score. The modified BDI-II score was calculated across the 18 remaining items, allowing for a maximum of 2 missing items that were imputed by the average score (total sample: 6.08%, main analysis sample: 4.47%). A dichotomous depression variable was calculated, with BDI scores > 13 indicating the presence of clinical symptoms of depression [56]. The internal consistency for the modified 18-item BDI score was satisfactory, with α = 0.92, ωh = 0.79 and ωt = 0.93. Reliability analyses showed that excluding the three items did not substantially reduce the internal consistency compared to the 21-item BDI score (S2 Table).

#### Socio-demographic variables

The present study includes socio-demographic information on gender (male, female), age, and socio-economic status, as these factors have been shown to be associated with adolescent sleep [58–60] as well as with depression [61, 62]. The question assessing “gender” likely captured both biological sex and gender identity. The item originally included a third answer option (“other”) that was excluded for the purpose of this analysis (only 0,8% reported “other gender”). Socio-economic status was measured using a proxy variable used in similar studies [63] that indicates self-perceived relative economic status (“Did you have enough money to be able to do the same things as your friends? If you think about the last week.“). Answers were given on a 1–5 Likert scale (*never*, *seldom*, *sometimes*, *often*, *always*), but were treated as a continuous variable in the analysis.

### Statistical analyses

Descriptive statistics are presented as the mean and standard deviation for all continuous variables and as frequencies and the median for categorial variables. Sleep parameters are reported for the main analysis sample of complete cases (*n* = 8449) and disaggregated by gender and for each of the two levels of depression. T-tests and chi-square tests were performed to assess differences between boys and girls and the depressed and non-depressed group. Bivariate correlations between sleep variables and BDI-II scores were analyzed.

Binary logistic regression analysis was performed to investigate the adjusted relationships between the selected sleep parameters and depression. As sleep duration was calculated based on bedtime, sleep onset latency, and wake time, there is inherently a high degree of collinearity between these variables. Only the following sleep variables were included in the regression model as they minimized problematic multicollinearity: sleep duration on weekdays, sleep duration on weekends, sleep quality (average sleep quality index score), and chronotype. The dependent variable of the regression model was BDI-II dichotomized as score ≤ 13 vs. BDI-II score > 13. The adjusted model encompassed the control variables gender, age, and self-perceived socio-economic status. For the regression analysis, listwise deletion was applied and therefore all results are reported for complete cases (*n* = 8449). Descriptive statistics for the sleep variables in the total sample (*n* = 10288) are available in the supporting information (S9–S12 Tables).

We conducted the logistic regression in a hierarchical fashion, entering the predictor variables as priori determined steps based on theoretical assumptions: 1) control variables, 2) Sleep duration on weekdays and weekends, 3) Sleep quality, 4) Chronotype. Results are only reported for the model including all steps, because the hierarchical regression did not influence the results. Unadjusted odds ratios were obtained from univariate regression models and indicate the bivariate effect for each predictor variable (Table 3, Bivariate effects).

Sensitivity analyses were conducted for the adjusted model to assess whether excluding the BDI-II items “change in sleep patterns”, “tiredness or fatigue”, “loss of sexual interest” from the BDI-II scores had an impact on the results. Sensitivity analyses were also conducted for the sleep quality index excluding the items “nightmares” and “premature awakenings”. Finally, we assessed whether excluding outliers on the bedtime and wake time variables affected the results.

Our models assumed a linear and normal distribution of effect. To ensure that linearity and normality were reasonable model assumptions, we also ran sensitivity tests with cubic and quadratic terms for the sleep duration variable; however, those models did not result in better model fit indices and were disregarded in future analyses.

A two-tailed 5% significance level was used. All statistical analyses were conducted in IBM SPSS Statistics (28.0.1.1). Reliability analyses were conducted in R Studio (RStudio 2022.12.0).

## Results

**Fig 1:**
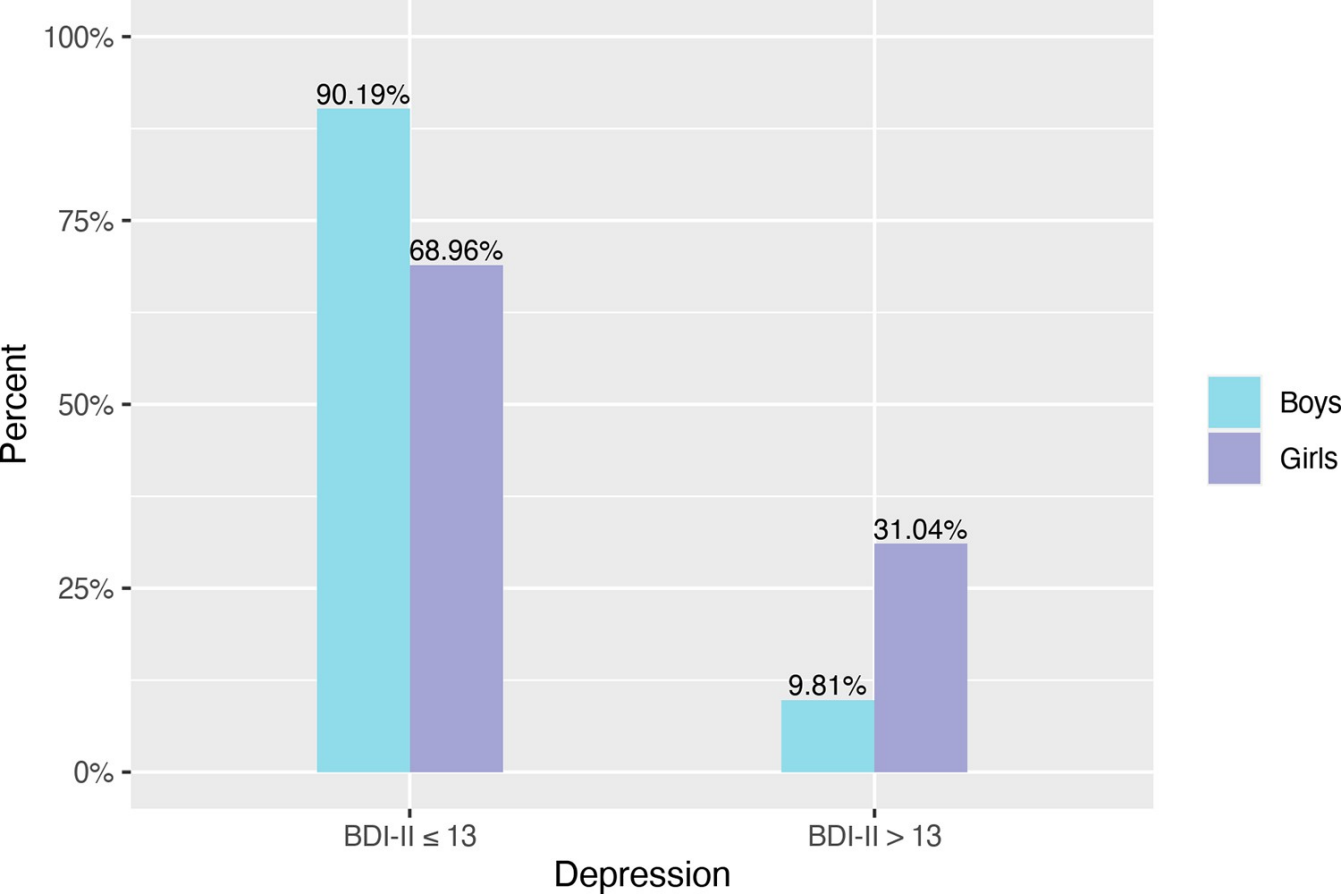
Relative frequency of depression in boys and girls. BDI-II scores > 13 indicate clinical levels of depression.

Clinical levels of depression were prevalent in a total of 20.6% of adolescents and were significantly more common in girls than in boys, *χ*^2^ = 581.986, p < .0001. The proportion of boys and girls with clinical levels of depression (BDI-II score > 13) and without depression (BDI-II score ≤ 13) is shown in Fig 1.

Bivariate correlations and descriptive statistics comparing sleep habits and sleep quality among boys and girls are shown in the supporting information (S3–S5 Tables). Sleep duration on weekdays was on average 8:05h (*SD* = 1:08h) in boys and 7:41h (*SD* = 1:13h) in girls. There was a shift towards later bedtimes, sleep onset times and wake times from weekdays to weekends. The average bedtime in the total sample changed from 22:26 (*SD* = 0:56) on weekdays to 24:08 (*SD* = 1:33) on weekends, and the sleep onset time from 22:55 (*SD* = 1:07) to 24:35 (*SD* = 1:42). Most notably, wake times were 3:06h later on weekends (*M* = 9:54, *SD* = 1:25) than on weekdays (*M* = 6:48, *SD* = 0:31). These changes in sleep patterns resulted in the average sleep duration for the total sample being 1:25h longer on weekends than on weekdays. Average sleep duration on weekends was 9:21h in boys (*SD* = 1:34) and 9:15h (*SD* = 1:34) in girls.

The data suggested that short (<8h) sleep duration on weekdays was highly common in the age group of 12–16-year-olds (Table 2). In our final sample (*n* = 8449), 45.6% of adolescents slept less than the recommended number of at least 8 hours per night; and 53.0% slept 8–10 hours, which is in accordance with the recommendations [21]. Long sleep duration is not common on weekdays, with only 1.4% of participants sleeping longer than 10 hours per night. Sleep duration less than 8 hours on weekdays is significantly more common in girls (52.8%) compared to boys (38.3%), while long sleep duration (more than 10 hours) is significantly more common in boys (1.9%) than in girls (0.8%) but overall, less prevalent for both groups than short sleep duration. On weekends, short sleep duration (less than 8 hours) is present in only 16.7% of adolescents. Sleep duration differs between boys and girls, but only for sleep duration times of 11 or more hours (boys: 12.6% vs. girls: 10.3%).

**Table 2:** Proportion of adolescents in different sleep duration categories, N (%).

	Total (n = 8449)	Boys (n = 4158)	Girls (n = 4291)	*χ* ^2^
**Sleep duration weekdays**		*χ*^2^ = 200.878, p < .001*		
Less than 6 hours	560 (6.6)	192 (4.6)	368 (8.6)	residual: -7.3*
6 or more hours, less than 7 hours	960 (11.4)	391 (9.4)	569 (13.3)	residual: -5.6*
7 or more hours, less than 8 hours	2335 (27.6)	1009 (24.3)	1326 (30.9)	residual: -6.8*
Recommended: 8–10 hours per night	4480 (53.0)	2487 (59.8)	1993 (46.4)	residual: 12.3*
More than 10 hours, less than 11 hours	99 (1.2)	68 (1.6)	31 (0.7)	residual: 3.9*
11 hours or more	15 (0.2)	11 (0.3)	4 (0.1)	residual: 1.9
**Sleep duration weekends**		*χ*^2^ = 13.163, p = .022*		
Less than 6 hours	222 (2.6)	102 (2.5)	120 (2.8)	residual: -1.0
6 or more hours, less than 7 hours	363 (4.3)	177 (4.3)	186 (4.3)	residual: -0.2
7 or more hours, less than 8 hours	830 (9.8)	392 (9.4)	438 (10.2)	residual: -1.2
Recommended: 8–10 hours per night	4566 (54.0)	2224 (53.5)	2342 (54.6)	residual: -1.0
More than 10 hours, less than 11 hours	1503 (17.8)	738 (17.7)	765 (17.8)	residual: -0.1
11 hours or more	965 (11.4)	525 (12.6)	440 (10.3)	residual: 3.4*

*Note: χ*^2^ test comparing boys and girls. Post hoc analysis: residuals and pairwise z-test with Bonferroni adjustment.

*p < 0.05.

Descriptive statistics for sleep habits and sleep quality in the depressed and non-depressed group are presented in the supporting information (S6 Table). Statistically significant differences (p < .01) between depressed and non-depressed adolescents were found for all sleep variables and in both boys and girls. Adolescents with depression (*n* = 1740) slept on average 53 minutes less on weekdays than adolescents without depression (*n* = 6709, t = 24.659, p < .0001). On weekends, this difference was smaller, with the average sleep duration being 32 minutes shorter in adolescents with depression than among those without depression (t = 11.250, p < .0001).

**Fig 2:**
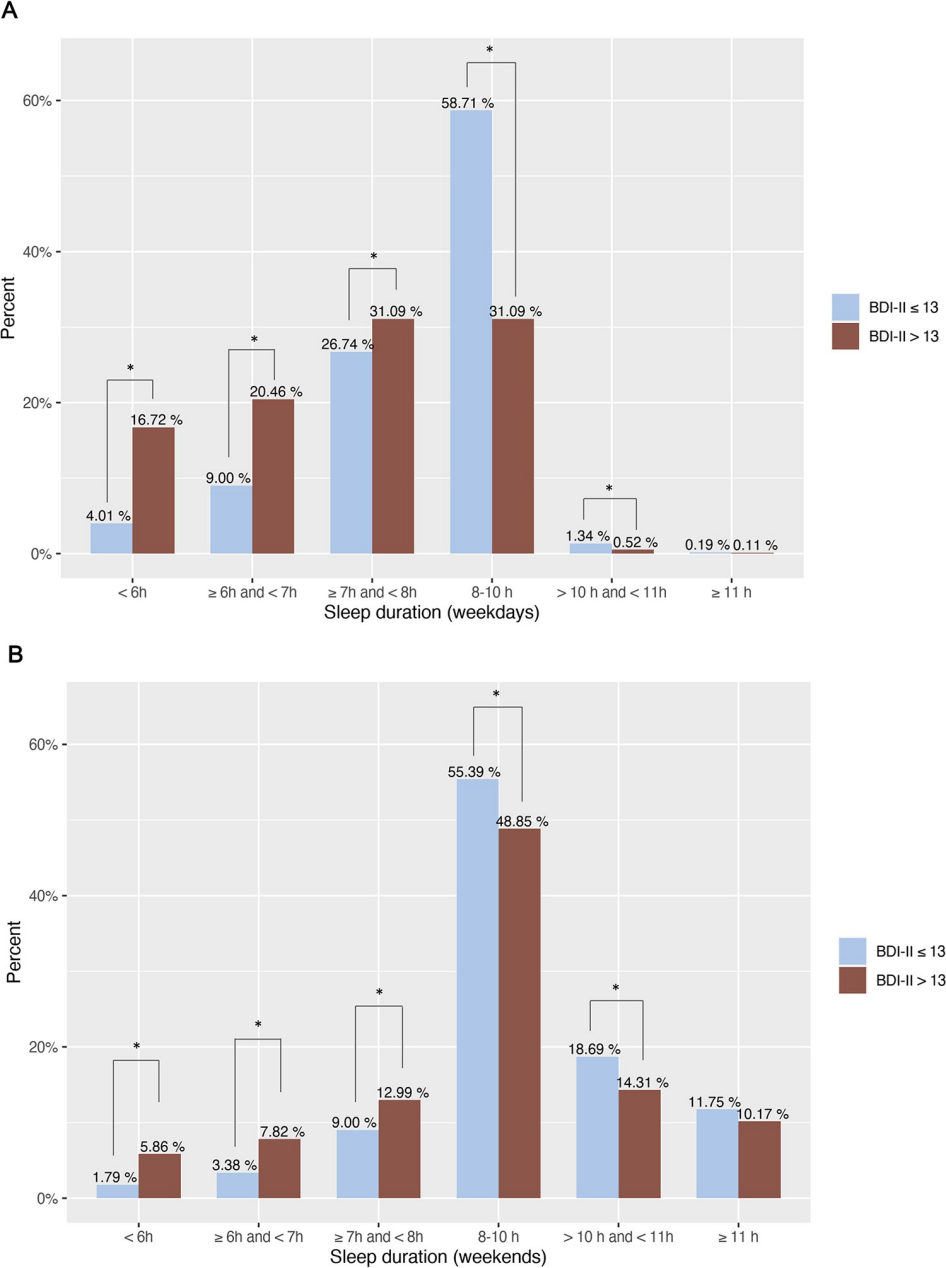
Sleep duration in depressed and non-depressed adolescents. A) Sleep duration on weekdays. B) Sleep duration on weekends. The bars display the percentage of adolescents in different categories of sleep duration. 8–10 hours is the recommended duration of sleep for this age group. * Proportions differ significantly from each other at the 0.05 level.

The frequency of different sleep duration categories among depressed and non-depressed adolescents is displayed in Fig 2. A substantial proportion of adolescents sleep less than the recommended 8 hours. Among adolescents with depression, only 31.09% meet the sleep recommendations on weekdays, while this percentage is 58.71% in adolescents without depression. Sleep duration times between 10-11h were less common in depressed adolescents, while no difference was found for times longer than 11h. On weekends, short sleep duration was much less prevalent: 14.17% of non-depressed and 26.67% depressed adolescents. Sleep duration times between 10-11h on weekends were less common in adolescents with depression, while no difference was found for times longer than 11h.

**Fig 3:**
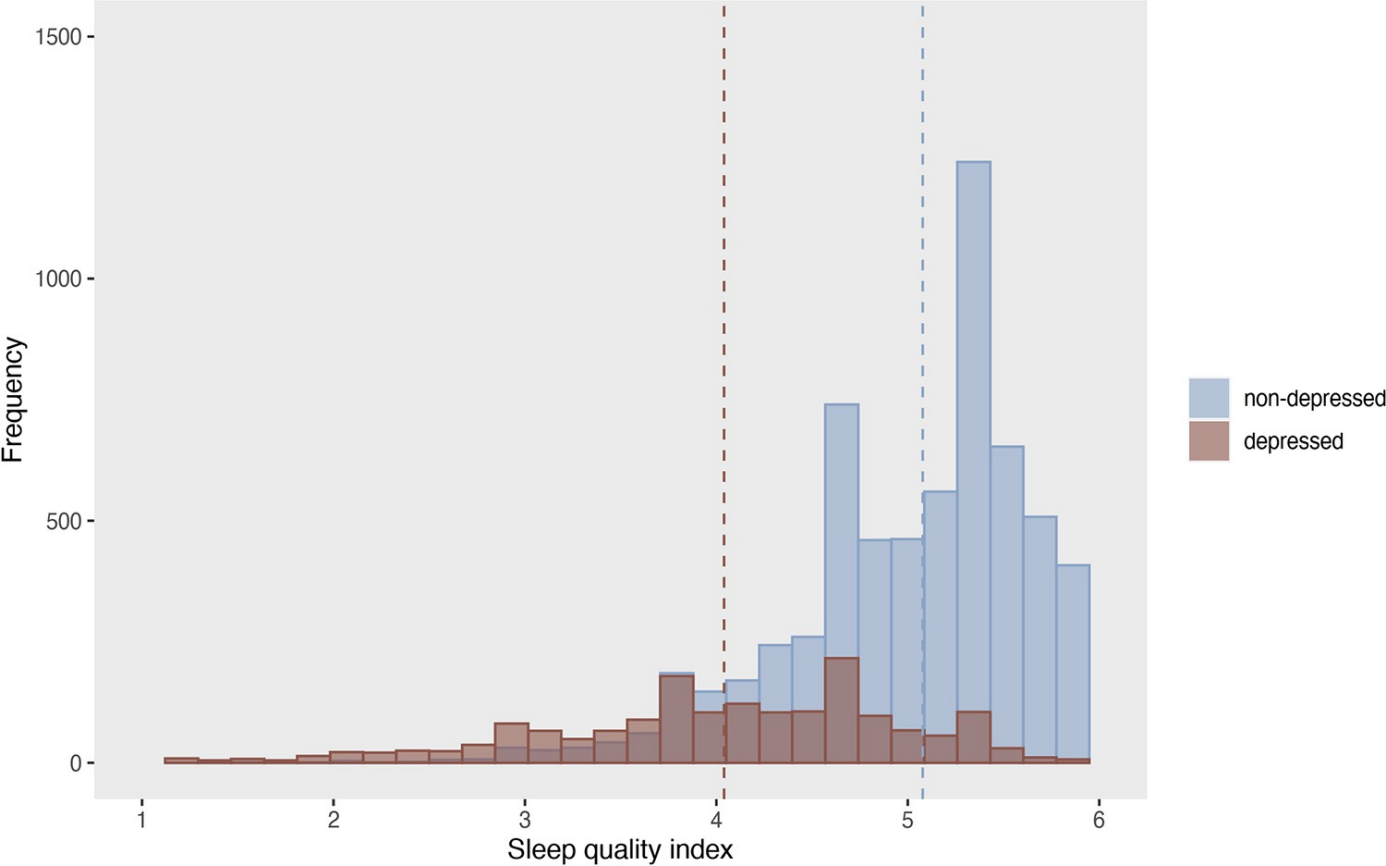
Sleep quality in depressed and non-depressed adolescents. Sleep quality index: range 1–6. The dashed line indicates the mean (non-depressed: 5.08, depressed: 4.04).

Sleep quality in the study sample, measured as the average score across 7 items on a 1–6 scale, had an average of *M* = 4.86 (*SD* = 0.84) (S5 Table). This was higher than the cut-off of 3, indicating that adolescents experienced self-perceived sleep problems on average less than three times per week and can be interpreted as “good” sleep quality. However, the average sleep quality index score was slightly but significantly lower in girls (*M* = 4.66, *SD* = 0.89, *n* = 4291) compared to boys (*M* = 5.08, *SD* = 0.72, *n* = 4158), t = 23.968, p < .0001. Further, sleep quality was significantly lower in the depressed group (*M* = 4.04, *SD* = 0.93, *n* = 1740) compared to the non-depressed group (*M* = 5.07, *SD* = 0.68, *n* = 6709, t = 43.680, p < .0001) (Fig 3). Descriptive statistics for the single sleep quality items are displayed in the supporting information (S5 and S6 Tables).

**Fig 4:**
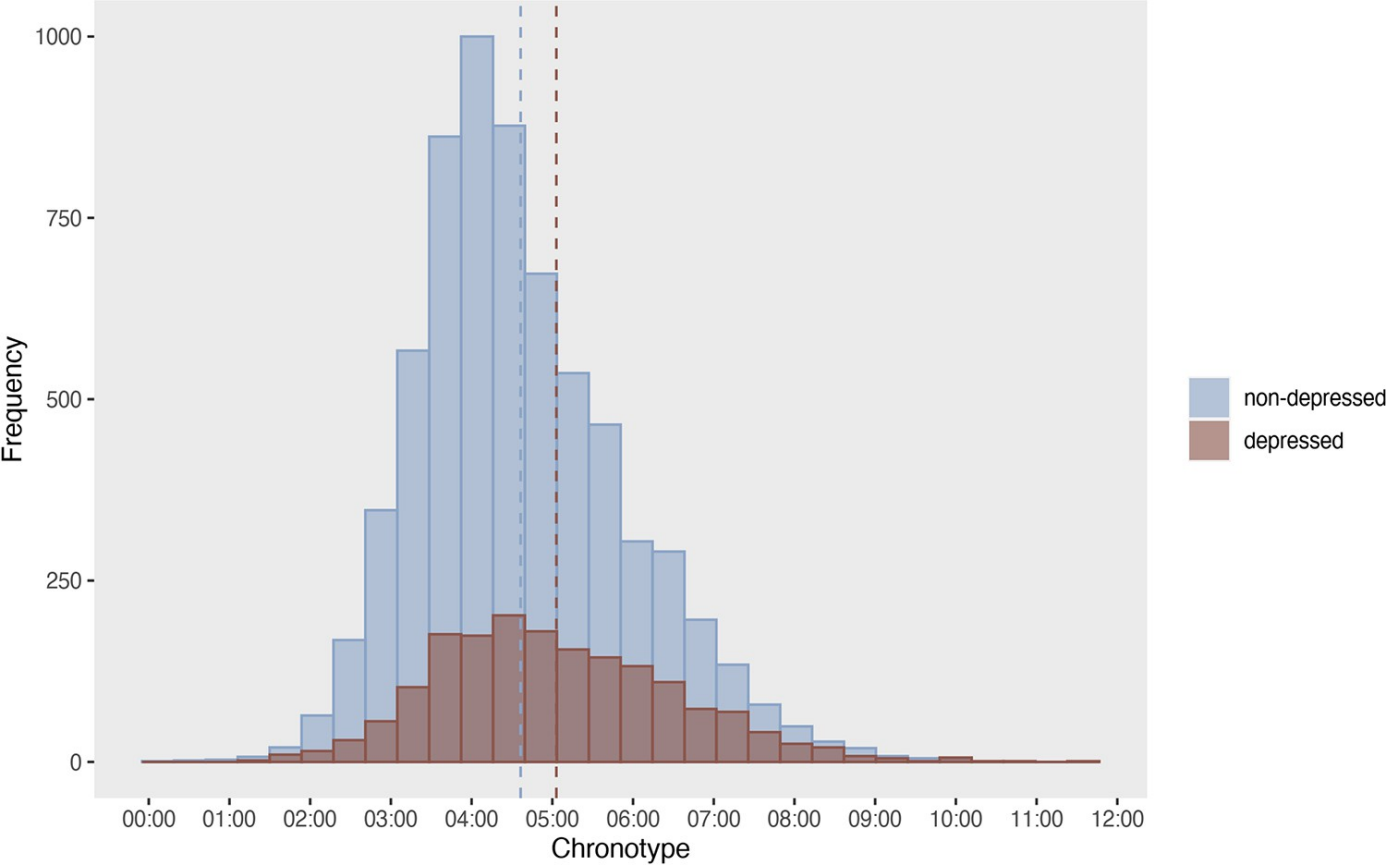
Chronotype in depressed and non-depressed adolescents. The dashed line indicates the mean midpoint of sleep on weekends, corrected for sleep debt (non-depressed: 4:36h, depressed: 5:03h).

Chronotype was 4:51 (*SD* = 1:22) in boys and 4:32 (*SD* = 1:17) in girls (t = 10.512, p < .0001) (S5 Table). Adolescents with depression had a later chronotype (*M* = 5:03, *SD* = 1:28) than adolescents without depression (*M* = 4:36, *SD* = 1:17, t = -11.429, p < .0001) (Fig 4).

Results from the adjusted regression model (Table 3) show that all predictor variables had a significant effect on depression, except for sleep duration on weekends. Girls were more likely to have clinical levels of depression (BDI-II scores > 13) than boys, with 215% higher odds of having depression (OR = 3.151, p < .0001). There was also a significant age effect, with a one SD-increase in age (equivalent to 0.7 years) being associated with an 8%-increase in the odds of depression (OR = 1.078, p = .0238). Higher self-perceived socioeconomic status (one SD equivalent to 0.9 scale points on a 1–5 scale) was associated with lower odds of depression (OR = 0.645, p < .0001). Sleep duration on weekdays significantly predicted depression. For every SD-increase in sleep duration (equivalent to 1 hour and 12 minutes), the odds of having depression decreased by 23% (OR = 0.773, p < .0001). Therefore, sleeping 30 minutes more on weekdays was associated with about 10% lower odds of having depression. Sleep duration on weekends however was not associated with depression (p = .9586). Perceived sleep quality, as indicated by the average sleep quality index score, was a significant predictor of depression. With a one SD-increase in sleep quality (equivalent to 0.9 score points on a 1–6 scale), the odds of having depression decreased by 67% (OR = 0.327, p < .0001). Later chronotypes, referring to individuals with a biological tendency for the sleep phase to take place at later times during a given 24-hour day, were found to have significantly higher odds of depression. Every SD-increase in chronotype (equivalent to 1 hour and 21 minutes) was associated with a 13% increase in the odds of having depression (OR = 1.126, p = .0017).

**Table 3:** Results of the logistic regression model.

Variable	Adjusted model				Bivariate effects	
	b	SE	p value	OR (95% CI)	OR (95% CI)	p value
Gender						
Girls	1.148*	.074	< .0001	3.151 (2.728–3.640)	3.942 (3.542–4.387)	< .0001
Boys	-1.148	.074	< .0001	0.317 (0.275–0.367)	0.242 (0.214–0.273)	< .0001
Age	0.075*	.033	.0238	1.078 (1.010–1.150)	1.130 (1.078–1.185)	< .0001
SES	-0.439*	.031	< .0001	0.645 (0.606–0.686)	0.541 (0.517–0.565)	< .0001
Sleep duration weekdays	-0.258*	.037	< .0001	0.773 (0.718–0.831)	0.490 (0.465–0.516)	< .0001
Sleep duration weekends	-0.002	.039	.9586	0.998 (0.925–1.077)	0.701 (0.666–0.737)	< .0001
Sleep quality	-1.119*	.040	< .0001	0.327 (0.302–0.353)	0.255 (0.239–0.273)	< .0001
Chronotype	0.119*	.038	.0017	1.126 (1.046–1.214)	1.367 (1.300–1.438)	< .0001

*Notes*: N = 8449 in the adjusted model (listwise deletion was applied). b = beta coefficient. SE = standard error. OR = odds ratio. CI = confidence interval.

Number of participants (n) analyzed in the bivariate effects: Gender: n = 9975, Age: n = 10026, SES: n = 10019, Sleep duration weekdays: n = 9315, Sleep duration weekends: n = 8950, Sleep quality: n = 9476, Chronotype: n = 8767.

The adjusted model shows the odds ratios for each variable adjusted for the remaining variables. Bivariate effects are displayed as the unadjusted odds ratios for each variable in a univariate regression model.

All continuous variables were z-standardized for the regression model. Therefore, odds ratios indicate the change in the odds of depression that is associated with a one standard deviation increase in the predictor variable.

*Statistically significant at p < .01

Sensitivity analyses for the adjusted model showed that excluding the items on sleep and sexual interest from the BDI score did not substantially affect the results (S7A Table). For the sleep quality index, sensitivity analyses confirmed that excluding the items on premature awakening and nightmares did not substantially change the effect of sleep quality on depression (S7B and S7C Table). Finally, trimming the bedtime and wake time variables did not substantially affect the results either (S7D Table).

## Discussion

This study investigated self-reported sleep habits and sleep quality and the association between selected sleep parameters (sleep duration, chronotype, sleep quality) and depression in 8449 Swedish adolescents between the age of 12 and 16. A substantial proportion of adolescents reported less than recommended sleep duration on weekdays, which was more common among girls than boys. Depression was significantly associated with shorter sleep duration, poorer sleep quality, and later chronotypes, emphasizing the importance of addressing sleep-related factors in understanding and managing depression among adolescents.

### Short sleep duration on weekdays

In this study, the average sleep duration on weekdays (7:53h) was below the recommended 8 hours of sleep per night for this age group [21]. Nearly half of the sample (45.6%) had sleep duration times shorter than 8 hours on weekdays, which is lower than a European study [24] that found 68% of 13–16-year-olds had short sleep duration. Additionally, 18.0% of adolescents in the present study slept less than 7 hours on weekdays, comparable to a Swedish study that found 12% of 12-13-year-old and 18% of 14-16-year-old adolescents had sleep duration times shorter than 7 hours [64]. The same study reported the average weekday sleep duration to be 7:56h, which is comparable to findings from our study. In a European study, weekday sleep duration was found to be 8:26h and 7:49h for 13 and 15-year-old Swedish adolescents, respectively [65]. However, the study was likely to overestimate actual sleep duration, as it was calculated solely based on self-reported bedtime and wake time, disregarding sleep-onset latency. Overall, these findings confirm that short sleep duration is a prevalent issue among 12-16-year-olds in Sweden and other countries around the world [3, 65].

In this study, statistically significant gender differences were found for all sleep variables, however, the effect-sizes for most gender differences were small. Further, the large sample size was well-powered to generate low p-values, thus the effect sizes should be interpreted rather literally. The most pronounced difference was found for sleep duration on weekdays, with girls sleeping 24 minutes less on average and having a higher prevalence (52.8%) of short sleep duration (i.e., <8h) compared to boys (38.3%). Evidence on gender differences in sleep duration is mixed, with some studies reporting that adolescent girls have shorter self-reported sleep duration on weekdays than boys [58, 59], while others found that girls sleep longer on weekdays than boys [66] or that there was no gender difference [67]. The mixed findings regarding gender differences in sleep duration suggested that further studies may be warranted.

### Compensation of sleep duration on weekends

On weekends, the average sleep duration was 9:18h, which is similar to previous studies from Sweden [64, 65] and consistent with findings from multiple countries showing that adolescents sleep longer on weekends than on weekdays [3]. In this study, long sleep duration (>10 hours) on weekdays was only prevalent in 1.4% of adolescents, compared to 29.2% on weekends. It is possible that adolescents have extended their sleep duration on weekends to compensate for short sleep duration on weekdays, a phenomenon referred to as weekend catch-up sleep [68]. This finding is quite noteworthy, because it may suggest that the vast majority of adolescents are unable to sleep enough during weekdays and are forced to compensate through weekend catch-up sleep. It’s possible that this catch-up sleep partially mitigates the adverse effects of the low weekday sleep and to an extent reduces the risk of depression [69]. However, it’s unlikely that it compensates for the cumulative effects of sleep loss, and irregular sleep habits may also contribute to additional negative health effects [68, 70].

While girls had shorter sleep duration times on weekends than boys (by 6 minutes), there was no difference in the prevalence of short sleep duration (<8 hours), but there was a higher prevalence of long sleep duration (>11 hours) in boys compared to girls. These findings were in contrast to previous studies showing that girls had longer sleep duration times on weekends than boys [58, 65, 66] and that girls have a higher prevalence of long sleep duration (>10h) on weekends than boys [71].

### Chronotype

Chronotype is the expression of how an individual’s natural circadian rhythm is synchronized with the light-dark schedule and in this study was determined by measuring a person’s sleep midpoint on weekends, corrected for sleep debt.

The average chronotype in this study was 04:41. For an adolescent to receive the minimum recommended sleep duration of 8 hours in accordance with their circadian rhythm, they would have to fall asleep at 00:41 (chronotype minus 4h) and wake up at 08:41 (chronotype plus 4h). In Sweden and other European countries, school start times are usually between 8 and 9 a.m., although regional differences exist [72]. Notably, we found that this social schedule did not align with the adolescents’ chronotypes at all, as it would imply that adolescents need to wake up and start school with their inner biological clock still being set at night-time. Only about 7% of adolescents in this sample had an optimal chronotype that was compatible with school-start times that require waking up at 7 a.m. and that allowed them to receive at least 8 hours of sleep.

Along with extended sleep duration on weekends, there was a shift in the timing of sleep, characterized by delayed bedtimes (by 1:42h) and wake times (by 3:06h), which may be attributed to the reduced impact from social obligations, such as school start times or extracurricular activities. The difference in sleep timing on weekdays and weekends, reflecting the misalignment between an individual’s internal biological clock and their external social schedule, is known as social jet lag [73] and appears to be associated with daytime sleepiness and poor academic performance [74]. In other words, our study suggests that both academic performance and overall public health could be improved substantially in adolescents if societal norms, including school start times, were reconsidered and challenged.

### Sleep quality

The average sleep quality in this study sample (average score: 4.86; range: 1–6) was above the cut-off of 3 and not within the range of clinically relevant symptoms of insomnia. As such, self-perceived quality in this study sample can be considered good or at least acceptable, with most adolescents experiencing sleep difficulties on average seldomly or a few times per month. Girls experienced issues such as difficulties falling asleep and waking up, and not feeling well-rested on awakenings more frequently than boys, converging with previous research showing that girls tend to have worse sleep quality than boys [24, 75] and higher prevalence rates of insomnia [58].

### Relationship between sleep and depression

The prevalence of depression (as defined by the BDI with a cut-off greater than 13) in this sample of 12–16-year-old Swedish adolescents was 21%. Depression was significantly more common in girls compared to boys, a finding well-established in the literature [61].

Results from our regression analysis in this study confirmed findings of previous studies showing that there is a negative association between sleep duration on weekdays and depression in adolescents [43, 76]. We found that an increase in sleep duration on weekdays by 30 minutes was associated with about 10% lower odds of having depression. Similar results have been found in Chinese and US samples [43, 76]. Interestingly, Roberts and Duong [76] found that short sleep duration also predicted future depressive symptoms, whereas neither depressive symptoms nor major depression predicted short sleep duration, implicating a causal pathway from sleep to depression rather than the other way around. Reducing sleep problems on a societal level therefore seems to be an appropriate way of preventing affective disorders.

Weekend sleep duration had a significant association with depression in the unadjusted regression model, but this relationship was not significant after adjusting the model. This finding aligns with a previous study [77] but contradicts other studies [43, 44]. It seems possible that catching up on sleep on weekends also reduces the risk of depression to some extent, as it mitigates sleep deprivation accumulated during weekdays [68]. Some studies have shown that adolescents with more than 2 hours of catch-up sleep on weekends do have an increased risk of mood disorders, suicidality [78], and lower subjective well-being [70]. However, this does not necessarily imply that long catch-up sleep causes psychopathology–it could just be that psychopathology is associated with an extra need for catch-up sleep.

Sleep quality was the strongest sleep-related predictor of depression in our regression model, which is in line with a previous meta-analysis reporting that sleep quality had a stronger and more reliable association with depression than sleep latency and sleep duration [45]. Those results suggest that subjective perceptions of sleep might be more important in the context of depression than objective sleep patterns. This may be due to that sleep quality is affected by psychological processes and cognitive biases that are congruent with the disorder; for example, people with depression may have more negative perceptions of their sleep [27]. Another possible explanation is that people are better at estimating their own sleep quality (i.e., their perceived need for sleep) than methods that rely on participants reporting sleep onset and wake times.

However, this explanation might not be fully harmonious with our additional finding that late chronotype was associated with depression (OR = 1.126), even after adjusting for sleep duration and sleep quality. This finding rather suggests that chronotype is an independent predictor of depression which is closely related to the circadian rhythm and human biology, but not subjectively experienced.

## General discussion

The results in this study, combined with previous research, underline the importance of sleep for the mental health of adolescents. Although the causal mechanisms are not clearly understood, it seems likely that sleep plays a critical role in the development of depression (see for instance Roberts & Duong [76]). We found that the associations amongst different sleep parameters were at least moderate in size. For instance, our model showed that increasing sleep duration by only 30 minutes lowered the risk of depression by 10 percent. Considering that an increase in sleep duration and quality probably has a number of other positive health and development effects [79–81] and very few negative ones, it is important to identify factors that obstruct healthy sleep habits, and methods that promote sleep duration and sleep quality in adolescents. Although some individuals may suffer from clinical conditions, there is evidence to believe that many new cases of depression can be prevented by targeting subclinical sleep problems and their determinants in healthy populations [82].

Determinants may include social factors like early school start times, which may play a central role, especially for individuals that have a late chronotype. Studies in college and adult populations suggest that the misalignment between an individual’s chronotype and their external social schedule results in a range of sleep problems, including poor sleep quality, sleep debt, and daytime sleepiness, that have been shown to mediate the association between sleep behaviors (i.e., short sleep duration, late chronotype) and depression [83, 84]. In fact, delaying school start times has been shown to increase adolescents’ sleep duration and improve academic performance [85, 86]. Studies found that a 15-minute delay in school start times was associated with a 5-minute increase in sleep duration [87] and a 50-minute delay was associated with a 43-minute increase [88].

Additional behavioral factors may of course also play an important role in the relationship between sleep and depression, for example screen time. There are a number of studies suggesting that problematic internet use and/or frequent screen time is associated with various mental health problems [89]. Interestingly, there is some evidence that this association seems to be mediated by reduced sleep [18, 90, 91]. If this is the case, parental monitoring and normative restrictions on adolescents’ night-time use of screens may provide another avenue for improving the sleep habits of young people.

For adolescents with more severe sleep problems, individual-level interventions such as bright light exposure, exogenous melatonin substitutes, and cognitive and behavioral interventions appear to be effective treatment options [49]. Findings within the field of sleep and depression suggest that treatments and health promotion interventions that integrate both sleep and mental health-related components could be preferrable to single-module interventions. For example, CBT-I (Cognitive-behavioral therapy for insomnia) in adolescents has been shown to increase self-reported sleep duration and reduce sleep onset latency, in addition to depression levels [92].

### Strengths and limitations

This study benefits from the use of a large sample of adolescents, which is quite unique in the field of sleep research, as data on sleep duration and chronotype are often not routinely collected in national health surveys. The sampling strategy enrolled schools from different socioeconomic areas in Stockholm County, which contributes to the generalizability of the findings presented in this study.

The primary weakness of this study is its cross-sectional design, which makes it difficult to draw conclusions about causality. Further studies using longitudinal designs with moderation and mediation analyses could clarify the causal pathways between sleep parameters and depression.

Another limitation of this study is the use of self-report measures of sleep behaviors, which appear to overestimate sleep duration compared to actigraphy and polysomnography [93–95]. Self-report measures may, however, be more appropriate to measure sleep quality, which reflects subjectively perceived quality of sleep rather than objective sleep behavior (which we actually found to be a better predictor for depression than duration).

One limitation regarding the calculation of sleep duration is that wakefulness after sleep onset was not taken into account, because this parameter was not quantified in the KSQ. However, frequent awakenings during the night were assessed as one component of the sleep quality score.

Regarding the role of chronotype in the context of depression, this study provides new insights as it measures chronotype as the actual timing of sleep behavior (i.e., corrected midpoint of sleep on weekends) [15] rather than a preference for peak performance (often referred to as “morningness” and “eveningness”) [96], which has been the focus of most previous studies [46]. It cannot necessarily be assumed that these different operationalizations of chronotype are directly comparable. However, one study found that both measures are strongly correlated [97], which indicates that comparisons may be possible at least to some degree.

In this study, sleep quality was measured by an index based on items from the Karolinska Sleep Questionnaire [52]. This questionnaire has only been validated among adult populations and may have some shortcomings when used for adolescent research.

Finally, it is possible that the sleep behaviors and perceived quality of sleep in the study sample were influenced by seasonality, that is during which month of the year data were collected. Future studies may be warranted to explore these effects.

## Conclusions

The results of this study build on existing evidence about insufficient sleep (and sleep opportunities) among adolescents. The study shows that a substantial proportion of Swedish adolescents is affected by short sleep duration, which could probably be prevented. This study also contributes to evidence about the relationship between sleep problems/behaviors and depression in this age group, showing that both short weekday sleep duration, poor sleep quality, and late chronotype are associated with increased odds of depression.

## Supporting information

S1 Table Definition and calculation of sleep parameters. KSQ = Karolinska Sleep Questionnaire.
(DOCX)

S2 Table Reliability coefficients for sleep quality indices and BDI-II scores in the main analysis sample, using all items and excluding certain items. N = 8449 (sample from the regression analysis, complete cases). α = Cronbach’s alpha coefficient. ωh = McDonald’s omega. ωt = total omega.
(DOCX)

S3 Table Bivariate pearson correlations for weekday sleep variables in the main analysis sample. N = 8449 (sample from the regression analysis, complete cases). Depression: BDI-II scores as a continuous variable. ^a^weekdays. *Correlation is significant at the 0.01 level.
(DOCX)

S4 Table Bivariate pearson correlations for weekend sleep variables in the main analysis sample. N = 8449 (sample from the regression analysis, complete cases). Depression: BDI-II scores as a continuous variable. ^a^weekends. *Correlation is significant at the 0.01 level.
(DOCX)

S5 Table Sleep habits and sleep quality in girls and boys in the main analysis sample (n = 8449), M (SD). Main analysis sample = sample from the regression analysis (complete cases). Boys and girls are compared using t-tests. M = Mean. Mdn = Median. SD = Standard deviation. SE (M) = standard error of the mean. Sleep habits and duration in hh:mm format. Sleep quality: Average sleep quality index score, range 1–6 (higher scores indicate better sleep quality). P-value based on t-test, comparing boys and girls. ^a^ t-test for unequal variances. *p < 0.05.
(DOCX)

S6 Table Sleep habits and sleep quality in non-depressed (BDI-II ≤ 13) and depressed (BDI-II > 13) adolescents in the main analysis sample (n = 8449), by gender, M (SD). Main analysis sample = sample from the regression analysis (complete cases). Depressed and non-depressed groups are compared using t-tests. M = Mean. SD = Standard deviation. Mdn = Median. Sleep habits and duration in hh:mm format. Sleep quality: Average sleep quality index score, range 1–6 (higher scores indicate better sleep quality). BDI-II: modified Beck Depression Inventory-II (excluding three items), range 0–63. P-value based on t-test, comparing depressed and non-depressed. ^a^ t-test for unequal variances.
(DOCX)

S7 Table Sensitivity analyses for the adjusted logistic regression model. (DOCX)

S8 Table STROBE checklist. (DOCX)

S9 Table Bivariate Pearson correlations for weekday sleep variables in the total sample. N = 10288 (total sample of participants with baseline data, aged 12–16 years old). Depression: BDI-II scores as a continuous variable. ^a^weekdays. *Correlation is significant at the 0.01 level.
(DOCX)

S10 Table Bivariate Pearson correlations for weekend sleep variables in the total sample. N = 10288 (total sample of participants with baseline data, aged 12–16 years old). Depression: BDI-II scores as a continuous variable. ^a^weekends. *Correlation is significant at the 0.01 level.
(DOCX)

S11 Table Sleep habits and sleep quality in girls and boys in the total sample (n = 10288), M (SD). Total sample = Participants with baseline data, aged 12–16 years old. Boys and girls are compared using t-tests. M = Mean. Mdn = Median. SD = Standard deviation. SE (M) = standard error of the mean. Sleep habits and duration in hh:mm format. Sleep quality: Average sleep quality index score, range 1–6 (higher scores indicate better sleep quality). P-value based on t-test, comparing boys and girls. ^a^ t-test for unequal variances. *p < 0.05.
(DOCX)

S12 Table Sleep habits and sleep quality in non-depressed (BDI-II ≤ 13) and depressed (BDI-II > 13) adolescents in the total sample (n = 10288), by gender, M (SD). Total sample = Participants with baseline data, aged 12–16 years old. Depressed and non-depressed groups are compared using t-tests. M = Mean. SD = Standard deviation. Mdn = Median. Sleep habits and duration in hh:mm format. Sleep quality: Average sleep quality index score, range 1–6 (higher scores indicate better sleep quality). BDI-II: modified Beck Depression Inventory-II (excluding three items), range 0–63. P-value based on t-test, comparing depressed and non-depressed. ^a^ t-test for unequal variances.
(DOCX)
