# Supplementary material for: Associations between sleep habits, quality, chronotype and depression in a large cross-sectional sample of Swedish adolescents
Source: PLoS One. 2023 Nov 2;18(11):e0293580. doi: 10.1371/journal.pone.0293580 (PMC10621812; doi:10.1371/journal.pone.0293580)
Supplement: S1 Table — KSQ = Karolinska Sleep Questionnaire. (DOCX) [file pone.0293580.s001.docx]

**S1 Table. Definition and calculation of sleep parameters**

| **Variable** | **Definition** | **Assessment method** |
| --- | --- | --- |
| Bedtime | “What time do you usually go to bed (turn off the light) during the school week/weekend?” | KSQ item |
| Sleep onset latency | “How long are you awake before you fall asleep (after you turn off the light) during the school week/weekend?” | KSQ item |
| Sleep onset time (SOT) | Bedtime plus sleep onset latency | Bedtime + Sleep onset latency |
| Wake time | “What time do you usually wake up during the school week/weekend?” | KSQ item |
| Sleep duration (TST) | Time between sleep onset time and wake time | Wake time - Sleep onset time |
| Time in bed | Time between bedtime and wake time | Wake time - Bedtime |
| Chronotype | Corrected midpoint of sleep on weekends/free days (MSF_sc_) | If TST_weekends_ ≤ TST_weekdays_:  MSF_sc_ = MSF = SOT_weekends_ + TST_weekends_/2  If TST_weekends_ > TST_weekdays_:  MSF_sc_ = MSF - (TST_weekends_ - TST_weeklyaverage_)/2 = SOT_weekends_ + TST_weeklyaverage_/2 |
| Sleep quality | Self-perceived quality of sleep | 7 KSQ items;  Average score (range 1-6) |

*Note:* KSQ = Karolinska Sleep Questionnaire.
