# Supplementary material for: Associations between sleep habits, quality, chronotype and depression in a large cross-sectional sample of Swedish adolescents
Source: PLoS One. 2023 Nov 2;18(11):e0293580. doi: 10.1371/journal.pone.0293580 (PMC10621812; doi:10.1371/journal.pone.0293580)
Supplement: S2 Table — N = 8449 (sample from the regression analysis, complete cases). α = Cronbach’s alpha coefficient. ωh = McDonald’s omega. ωt = total omega. (DOCX) [file pone.0293580.s002.docx]

**S2 Table. Reliability coefficients for sleep quality indices and BDI-II scores in the main analysis sample, using all items and excluding certain items.**

| **Variable** | **α** | **ωh** | **ωt** |
| --- | --- | --- | --- |
| **Sleep quality items and indices** |  |  |  |
| Including all 7 items | 0.79 | 0.65 | 0.85 |
| Excluding ’Difficulties falling sleep | 0.75 | 0.70 | 0.85 |
| Excluding ’Difficulties waking up’ | 0.78 | 0.69 | 0.85 |
| Excluding ’Repeated awakenings with difficulties falling asleep again’ | 0.74 | 0.62 | 0.82 |
| Excluding ’Nightmares’ | 0.77 | 0.51 | 0.84 |
| Excluding ’Not well-rested on awakening’ | 0.75 | 0.72 | 0.84 |
| Excluding ’Premature awakenings’ | 0.78 | 0.65 | 0.86 |
| Excluding ’Disturbed/restless sleep’ | 0.74 | 0.52 | 0.81 |
| **BDI-II items and scale computations** |  |  |  |
| Including all 21 items | 0.92 | 0.79 | 0.93 |
| Excluding ’Changes in sleep patterns’ | 0.92 | 0.79 | 0.93 |
| Excluding ’Tiredness or fatigue’ | 0.91 | 0.81 | 0.92 |
| Excluding ’Loss of sexual interest’ | 0.92 | 0.79 | 0.93 |
| Excluding ’Changes in sleep patterns’ and ’Tiredness or fatigue’ | 0.91 | 0.81 | 0.92 |
| Excluding ’Changes in sleep patterns’ and ’Tiredness or fatigue’ and ’Loss of sexual interest’ | 0.92 | 0.82 | 0.93 |

*Note:* N = 8449 (sample from the regression analysis, complete cases).
α = Cronbach’s alpha coefficient. ωh = McDonald’s omega. ωt = total omega.
