# Supplementary material for: Associations between sleep habits, quality, chronotype and depression in a large cross-sectional sample of Swedish adolescents
Source: PLoS One. 2023 Nov 2;18(11):e0293580. doi: 10.1371/journal.pone.0293580 (PMC10621812; doi:10.1371/journal.pone.0293580)
Supplement: S3 Table — N = 8449 (sample from the regression analysis, complete cases). Depression: BDI-II scores as a continuous variable. aweekdays. *Correlation is significant at the 0.01 level. (DOCX) [file pone.0293580.s003.docx]

**S3 Table. Bivariate Pearson correlations for weekday sleep variables in the main analysis sample.**

|  | Depression | Bedtime^a^ | Sleep onset latency^a^ | Sleep onset time^a^ | Wake time^a^ | Sleep duration^a^ | Time in bed^a^ | Chronotype |
| --- | --- | --- | --- | --- | --- | --- | --- | --- |
| Bedtime^a^ | .224* | - |  |  |  |  |  |  |
| Sleep onset latency^a^ | .289* | .103* | - |  |  |  |  |  |
| Sleep onset time^a^ | .323* | .883* | .557* | - |  |  |  |  |
| Wake time^a^ | -.163* | .131* | -.044* | .089* | - |  |  |  |
| Sleep duration^a^ | -.374* | -.770* | -.542* | -.898* | .359* | - |  |  |
| Time in bed^a^ | -.291* | -.856* | -.118* | -.770* | .401* | .899* | - |  |
| Chronotype | .173* | .446* | .232* | .481* | .192* | -.367* | -.312* | - |
| Sleep quality | -.628* | -.251* | -.354* | -.376* | .100* | .397* | .284* | -.191* |

*Note:* N = 8449 (sample from the regression analysis, complete cases).
Depression: BDI-II scores as a continuous variable.
^a^weekdays

*Correlation is significant at the 0.01 level.
