# Supplementary material for: Associations between sleep habits, quality, chronotype and depression in a large cross-sectional sample of Swedish adolescents
Source: PLoS One. 2023 Nov 2;18(11):e0293580. doi: 10.1371/journal.pone.0293580 (PMC10621812; doi:10.1371/journal.pone.0293580)
Supplement: S4 Table — N = 8449 (sample from the regression analysis, complete cases). Depression: BDI-II scores as a continuous variable. aweekends. *Correlation is significant at the 0.01 level. (DOCX) [file pone.0293580.s004.docx]

**S4 Table. Bivariate Pearson correlations for weekend sleep variables in the main analysis sample.**

|  | Depression | Bedtime^a^ | Sleep onset latency^a^ | Sleep onset time^a^ | Wake time^a^ | Sleep duration^a^ | Time in bed^a^ | Chronotype |
| --- | --- | --- | --- | --- | --- | --- | --- | --- |
| Bedtime^a^ | .210* | - |  |  |  |  |  |  |
| Sleep onset latency^a^ | .189* | .094* | - |  |  |  |  |  |
| Sleep onset time^a^ | .256* | .938* | .432* | - |  |  |  |  |
| Wake time^a^ | .114* | .519* | .111* | .508* | - |  |  |  |
| Sleep duration^a^ | -.175* | -.549* | -.369* | -.626* | .354* | - |  |  |
| Time in bed^a^ | -.112* | -.553* | .009 | -.498** | .425* | .926* | - |  |
| Chronotype | .173* | .917* | .344* | .950* | .657* | -.437* | -.331* | - |
| Sleep quality | -.628* | -.217* | -.226* | -.275* | -.124* | .187* | .109* | -.191* |

*Note:* N = 8449 (sample from the regression analysis, complete cases).
Depression: BDI-II scores as a continuous variable.
^a^weekends

*Correlation is significant at the 0.01 level.
