# Supplementary material for: Associations between sleep habits, quality, chronotype and depression in a large cross-sectional sample of Swedish adolescents
Source: PLoS One. 2023 Nov 2;18(11):e0293580. doi: 10.1371/journal.pone.0293580 (PMC10621812; doi:10.1371/journal.pone.0293580)
Supplement: S5 Table — Main analysis sample = sample from the regression analysis (complete cases). Boys and girls are compared using t-tests. M = Mean. Mdn = Median. SD = Standard deviation. SE (M) = standard error of the mean. Sleep habits and duration in hh:mm format. Sleep quality: Average sleep quality index score, range 1–6 (higher scores indicate better sleep quality). P-value based on t-test, comparing boys and girls. a t-test for unequal variances. *p < 0.05. (DOCX) [file pone.0293580.s005.docx]

**S5 Table. Sleep habits and sleep quality in girls and boys in the main analysis sample (n=8449), M (SD).**

|  | **Total** (n=8449) | **SE (M)** | **Boys** (n=4158) | **Girls** (n=4291) | **p value** | **Mean difference (SE)** |
| --- | --- | --- | --- | --- | --- | --- |
| **Weekdays** |  |  |  |  |  |  |
| Bedtime | 22:26 (0:56) | 0:00 | 22:24 (0:56) | 22:28 (0:56) | p = .0002, t = -3.788 | -0:04 (0:01) |
| Sleep onset latency | 00:29 (0:32) | 0:00 | 00:27 (0:31) | 00:31 (0:32) | p < .0001^a^, t = -5.787 | -0:04 (0:01) |
| Sleep onset time | 22:55 (1:07) | 0:01 | 22:50 (1:06) | 22:59 (1:08) | p < .0001, t = -5.893 | -0:08 (0:01) |
| Wake time | 6:48 (0:31) | 0:00 | 6:56 (0:30) | 6:40 (0:30) | p < .0001^a^, t = 23.722 | 0:15 (0:00) |
| Sleep duration | 7:53 (1:11) | 0:01 | 8:05 (1:08) | 7:41 (1:13) | p < .0001^a^, t = 15.903 | 0:24 (0:01) |
| Time in bed | 8:21 (1:00) | 0:00 | 8:32 (0:57) | 8:11 (1:01) | p < .0001^a^, t = 15.721 | 0:20 (0:01) |
|  |  |  |  |  |  |  |
| **Weekends** |  |  |  |  |  |  |
| Bedtime | 24:08 (1:33) | 0:01 | 24:14 (1:36) | 24:01 (1:29) | p < .0001^a^, t = 6.094 | 0:12 (0:02) |
| Sleep onset latency | 00:28 (0:36) | 0:00 | 00:27 (0:35) | 00:29 (0:37) | p = .0025, t = -3.025 | -0:02 (0:01) |
| Sleep onset time | 24:35 (1:42) | 0:01 | 24:40 (1:44) | 24:31 (1:41) | p = .0001^a^, t = 4.466 | 0:09 (0:02) |
| Wake time | 9:54 (1:25) | 0:01 | 10:02 (1:28) | 9:46 (1:22) | p < .0001^a^, t = 8.767 | 0:16 (0:01) |
| Sleep duration | 9:18 (1:34) | 0:01 | 9:21 (1:34) | 9:15 (1:34) | p = .0022, t = 3.065 | 0:06 (0:02) |
| Time in bed | 9:46 (1:27) | 0:01 | 9:48 (1:29) | 9:44 (1:26) | p = .0385, t = 2.070 | 0:03 (0:01) |
|  |  |  |  |  |  |  |
| **Chronotype** | 4:41 (1:20) | 0:01 | 4:51 (1:22) | 4:32 (1:17) | p < .0001^a^, t = 10.512 | 0:18 (0:01) |
| **Sleep quality** | 4.86 (0.84) | .009 | 5.08 (0.72) | 4.66 (0.89) | p < .0001^a^, t = 23.968 | 0.42 (.018) |
| **Single items of the sleep quality index** | | | |  |  |  |
| 1. Difficulties  falling asleep | 4.59 (1.36) Mdn: 5.00 | .015 | 4.87 (1.21) Mdn: 5.00 | 4.32 (1.44) Mdn: 5.00 | p < .0001^a^, t = 19.178 | 0.55 (.029) |
| 2. Difficulties  waking up | 4.14 (1.65) Mdn: 5.00 | .018 | 4.41 (1.59) Mdn: 5.00 | 3.87 (1.68) Mdn: 4.00 | p < .0001^a^, t = 14.937 | 0.53 (.036) |
| 3. Repeated awakenings with difficulties falling asleep again | 5.23 (1.10) Mdn: 6.00 | .012 | 5.39 (0.98) Mdn: 6.00 | 5.08 (1.19) Mdn: 5.00 | p < .0001^a^, t = 13.151 | 0.31 (.024) |
| 4. Nightmares | 5.39 (0.96) Mdn: 6.00 | .010 | 5.58 (0.78)  Mdn: 6.00 | 5.21 (1.07) Mdn: 6.00 | p < .0001^a^, t = 18.072 | 0.37 (.020) |
| 5. Not well-rested on awakenings | 4.28 (1.58) Mdn: 5.00 | .017 | 4.59 (1.46)  Mdn: 5.00 | 3.99 (1.65) Mdn: 4.00 | p < .0001^a^, t = 17.814 | 0.60 (.034) |
| 6. Premature awakenings | 5.03 (1.16) Mdn: 5.00 | .013 | 5.13 (1.08) Mdn: 5.00 | 4.92 (1.22) Mdn: 5.00 | p < .0001^a^, t = 8.346 | 0.21 (.025) |
| 7. Disturbed/ restless sleep | 5.38 (1.04)  Mdn: 6.00 | .011 | 5.58 (0.85)  Mdn: 6.00 | 5.20 (1.16) Mdn: 6.00 | p < .0001^a^, t = 17.172 | 0.38 (.022) |

*Note:* Main analysis sample = sample from the regression analysis (complete cases).
Boys and girls are compared using t-tests. M = Mean. Mdn = Median. SD = Standard deviation. SE (M) = standard error of the mean. Sleep habits and duration in hh:mm format.
Sleep quality: Average sleep quality index score, range 1-6 (higher scores indicate better sleep quality).
P-value based on t-test, comparing boys and girls.

^a^ t-test for unequal variances.

*p < 0.05.
