# Supplementary material for: Associations between sleep habits, quality, chronotype and depression in a large cross-sectional sample of Swedish adolescents
Source: PLoS One. 2023 Nov 2;18(11):e0293580. doi: 10.1371/journal.pone.0293580 (PMC10621812; doi:10.1371/journal.pone.0293580)
Supplement: S6 Table — Main analysis sample = sample from the regression analysis (complete cases). Depressed and non-depressed groups are compared using t-tests. M = Mean. SD = Standard deviation. Mdn = Median. Sleep habits and duration in hh:mm format. Sleep quality: Average sleep quality index score, range 1–6 (higher scores indicate better sleep quality). BDI-II: modified Beck Depression Inventory-II (excluding three items), range 0–63. P-value based on t-test, comparing depressed and non-depressed. a t-test for unequal variances. (DOCX) [file pone.0293580.s006.docx]

**S6 Table. Sleep habits and sleep quality in non-depressed (BDI-II ≤ 13) and depressed (BDI-II > 13) adolescents in the main analysis sample (n=8449), by gender, M (SD).**

|  | **Total** (n=8449) | | |  | **Boys** (n=4158) | | |  | **Girls** (n=4291) | | |
| --- | --- | --- | --- | --- | --- | --- | --- | --- | --- | --- | --- |
|  | **BDI**  **≤ 13**  (n=6709) | **BDI  > 13**  (n=1740) | **P value** |  | **BDI  ≤ 13** (n=3750) | **BDI  > 13** (n=408) | **P value** |  | **BDI  ≤ 13** (n=2959) | **BDI  > 13** (n=1332) | **P value** |
| **Weekdays** | | | | | | | | | | | |
| Bedtime | 22:21 (0:52) | 22:45 (1:04) | p < .0001^a^, t = -14.567 |  | 22:22 (0:54) | 22:42  (1:05) | p < .0001^a^, t = -6.198 |  | 22:20  (0:50) | 22:46  (1:03) | p < .0001^a^, t = -13.108 |
| Sleep onset latency | 0:25 (0:27) | 0:43 (0:42) | p < .0001^a^, t = -17.261 |  | 0:25 (0:29) | 0:45  (0:44) | p < .0001^a^, t = -9.082 |  | 0:25  (0:25) | 0:43  (0:41) | p < .0001^a^, t = -14.265 |
| Sleep onset time | 22:46  (1:01) | 23:28  (1:17) | p < .0001^a^, t = -21.277 |  | 22:46  (1:03) | 23:27  (1:18) | p < .0001^a^, t = -10.225 |  | 22:45  (0:58) | 23:29  (1:17) | p < .0001^a^, t = -18.297 |
| Wake time | 6:50  (0:30) | 6:39  (0:35) | p < .0001^a^, t = 11.351 |  | 6:56  (0:30) | 6:52  (0:33) | p = .0171^a^, t = 2.392 |  | 6:42  (0:28) | 6:36  (0:35) | p < .0001^a^, t = 5.848 |
| Sleep duration | 8:04 (1:04) | 7:11  (1:23) | p < .0001^a^, t = 24.659 |  | 8:10 (1:04) | 7:24  (1:22) | p < .0001^a^, t = 10.691 |  | 7:56  (1:02) | 7:06  (1:23) | p < .0001^a^, t = 19.442 |
| Time in bed | 8:29 (0:55) | 7:54  (1:10) | p < .0001^a^, t = 19.030 |  | 8:34  (0:56) | 8:09  (1:09) | p < .0001^a^, t = 7.022 |  | 8:21  (0:54) | 7:49  (1:10) | p < .0001^a^, t = 14.831 |
| **Weekends** | | | | | | | | | | | |
| Bedtime | 24:00 (1:29) | 24:37  (1:41) | p < .0001^a^, t = -13.905 |  | 24:10 (1:33) | 24:50  (1:49) | p < .0001^a^, t = -7.144 |  | 23:47  (1:21) | 24:33  (1:39) | p < .0001^a^, t = -14.794 |
| Sleep onset latency | 0:25 (0:32) | 0:39 (0:45) | p < .0001^a^, t = -11.946 |  | 0:25 (0:33) | 0:40  (0:48) | p < .0001^a^, t = -5.965 |  | 0:25 (0:31) | 0:39  (0:45) | p < .0001^a^, t = -10.239 |
| Sleep onset time | 24:25 (1:37) | 01:16  (1:53) | p < .0001^a^, t = -17.196 |  | 24:35  (1:41) | 01:30 (1:54) | p < .0001^a^, t = -9.230 |  | 24:12  (1:29) | 01:12  (1:52) | p < .0001^a^, t = -17.058 |
| Wake time | 9:50 (1:22) | 10:08  (1:36) | p < .0001^a^, t = -7.343 |  | 9:59  (1:26) | 10:25  (1:41) | p < .0001^a^, t = -4.850 |  | 9:38  (1:15) | 10:03  (1:34) | p < .0001^a^, t = -8.751 |
| Sleep duration | 9:24 (1:28) | 8:52  (1:51) | p < .0001^a^, t = 11.250 |  | 9:24  (1:31) | 8:54  (1:56) | p < .0001^a^, t = 4.897 |  | 9:25  (1:24) | 8:51  (1:49) | p < .0001^a^, t = 10.024 |
| Time in bed | 9:49  (1:23) | 9:31  (1:43) | p < .0001^a^, t = 6.934 |  | 9:49  (1:26) | 9:34  (1:50) | p < .0001^a^, t = 2.629 |  | 9:50  (1:18) | 9:30  (1:41) | p < .0001^a^, t = 6.409 |
| **Chronotype** | 4:36 (1:17) | 5:03  (1:28) | p < .0001^a^, t = -11.429 |  | 4:48  (1:21) | 5:20  (1:30) | p < .0001^a^, t = -6.945 |  | 4:21  (1:09) | 4:57  (1:27) | p < .0001^a^, t = -13.233 |
|  |  |  |  |  |  |  |  |  |  |  |  |
| **Sleep quality** | 5.08  (0.66) | 4.04  (0.93) | p < .0001^a^, t = 43.680 |  | 5.17  (0.63) | 4.21 (0.91) | p < .0001^a^, t = 21.029 |  | 4.96  (0.68) | 3.99  (0.93) | p < .0001^a^, t = 33.973 |
| **Single items of the sleep quality index** | | | |  |  |  |  |  |  |  |  |
| 1. Difficulties  falling asleep | 4.85  (1.16) Mdn: 5 | 3.60  (1.59)  Mdn: 4 | p < .0001^a^, t = 30.531 |  | 4.99  (1.10) Mdn: 5 | 3.84 (1.60)  Mdn: 4 | p < .0001^a^, t = 14.085 |  | 4.67  (1.22)  Mdn: 5 | 3.53  (1.59)  Mdn: 4 | p < .0001^a^, t = 23.406 |
| 2. Difficulties  waking up | 4.38  (1.55) Mdn: 5 | 3.19  (1.70)  Mdn: 3 | p < .0001^a^, t = 26.491 |  | 4.52  (1.53) Mdn: 5 | 3.35 (1.71)  Mdn: 3 | p < .0001^a^, t = 13.319 |  | 4.21  (1.56)  Mdn: 5 | 3.15  (1.69)  Mdn: 3 | p < .0001^a^, t = 19.431 |
| 3. Repeated awakenings with difficulties falling asleep again | 5.43  (0.88)  Mdn: 6 | 4.48  (1.48)  Mdn: 5 | p < .0001^a^, t = 25.590 |  | 5.49  (0.84)  Mdn: 6 | 4.50 (1.53)  Mdn: 5 | p < .0001^a^, t = 12.855 |  | 5.35  (0.91)  Mdn: 6 | 4.47  (1.47)  Mdn: 5 | p < .0001^a^, t = 20.205 |
| 4. Nightmares | 5.55  (0.75)  Mdn: 6 | 4.79  (1.34)  Mdn: 5 | p < .0001^a^, t = 22.468 |  | 5.64  (0.68)  Mdn: 6 | 5.00 (1.29)  Mdn: 5 | p < .0001^a^, t = 9.916 |  | 5.43  (0.82)  Mdn: 6 | 4.73  (1.36) Mdn: 5 | p < .0001^a^, t = 17.368 |
| 5. Not well-rested on awakenings | 4.58  (1.42)  Mdn: 5 | 3.14  (1.67)  Mdn: 3 | p < .0001^a^, t = 33.004 |  | 4.71  (1.38)  Mdn: 5 | 3.45 (1.65)  Mdn: 4 | p < .0001^a^, t = 14.930 |  | 4.41  (1.45)  Mdn: 5 | 3.05  (1.67) Mdn: 3 | p < .0001^a^, t = 25.824 |
| 6. Premature awakenings | 5.16  (1.02)  Mdn: 5 | 4.50  (1.47)  Mdn: 5 | p < .0001^a^, t = 17.765 |  | 5.19  (1.01) Mdn: 5 | 4.57  (1.43)  Mdn: 5 | p < .0001^a^, t = 8.561 |  | 5.12  (1.03)  Mdn: 5 | 4.48  (1.48) Mdn: 5 | p < .0001^a^, t = 14.448 |
| 7. Disturbed/ restless sleep | 5.59  (0.76)  Mdn: 6 | 4.57  (1.47)  Mdn: 5 | p < .0001^a^, t = 28.052 |  | 5.67  (0.71)  Mdn: 6 | 4.73  (1.43)  Mdn: 5 | p < .0001^a^, t = 13.083 |  | 5.50  (0.82)  Mdn: 6 | 4.53  (1.48) Mdn: 5 | p < .0001^a^, t = 22.586 |

*Note:* Main analysis sample = sample from the regression analysis (complete cases).
Depressed and non-depressed group are compared using t-tests.
M = Mean. SD = Standard deviation. Mdn = Median. Sleep habits and duration in hh:mm format.
Sleep quality: Average sleep quality index score, range 1-6 (higher scores indicate better sleep quality).

BDI-II: modified Beck Depression Inventory-II (excluding three items), range 0-63.

P-value based on t-test, comparing depressed and non-depressed.

^a^ t-test for unequal variances.
