# Supplementary material for: Associations between sleep habits, quality, chronotype and depression in a large cross-sectional sample of Swedish adolescents
Source: PLoS One. 2023 Nov 2;18(11):e0293580. doi: 10.1371/journal.pone.0293580 (PMC10621812; doi:10.1371/journal.pone.0293580)
Supplement: S7 Table — (DOCX) [file pone.0293580.s007.docx]

**S7 Table. Sensitivity analyses for the adjusted logistic regression model.**

**S7A Table. Regression results when including the 3 items from the BDI-II score.**

| **Variable** | **b** | **SE** | **p value** | **OR  (95% CI)** |
| --- | --- | --- | --- | --- |
| Gender^a^ | 1.109* | .074 | < .0001 | 3.031 (2.621 – 3.505) |
| Age | 0.086* | .034 | .0103 | 1.090 (1.021 – 1.164) |
| SES | -0.456* | .032 | < .0001 | 0.634 (0.595 – 0.674) |
| Sleep duration weekdays | -0.268* | .038 | < .0001 | 0.765 (0.710 – 0.824) |
| Sleep duration weekends | 0.019 | .039 | .6288 | 1.019 (0.944 – 1.101) |
| Sleep quality | -1.209* | .041 | < .0001 | 0.298 (0.275 – 0.324) |
| Chronotype | 0.160* | 0.038 | < .0001 | 1.174 (1.089 – 1.266) |

*Note:* Sensitivity analysis was conducted for the adjusted regression model. b = beta coefficient. SE = standard error. OR = odds ratio. CI = confidence interval.
Items that were excluded from the BDI-II score in the primary model: “Changes in sleep patterns”, “Tiredness or fatigue”, “Loss of sexual interest”.

^a^Gender=girls (compared to boys)

*Statistically significant at p<.01

**S7B Table. Regression results when excluding the sleep quality item *premature awakenings*.**

| **Variable** | **b** | **SE** | **p value** | **OR  (95% CI)** |
| --- | --- | --- | --- | --- |
| Gender^a^ | 1.112* | .073 | < .0001 | 3.039 (2.632 – 3.509) |
| Age | 0.072* | .033 | .0306 | 1.074 (1.007 – 1.146) |
| SES | -0.445* | .031 | < .0001 | 0.641 (0.603 – 0.681) |
| Sleep duration weekdays | -0.244* | .038 | < .0001 | 0.784 (0.728 – 0.844) |
| Sleep duration weekends | -0.048 | .039 | .2118 | 0.953 (0.883 – 1.028) |
| Sleep quality | -1.114* | .040 | < .0001 | 0.328 (0.304 – 0.355) |
| Chronotype | 0.077* | .038 | .0437 | 1.080 (1.002 – 1.164) |

*Note:* Sensitivity analysis was conducted for the adjusted regression model. b = beta coefficient. SE = standard error. OR = odds ratio. CI = confidence interval.

^a^Gender=girls (compared to boys)

*Statistically significant at p<.01

**S7C Table. Regression results when excluding the sleep quality item *nightmares*.**

| **Variable** | **b** | **SE** | **p value** | **OR  (95% CI)** |
| --- | --- | --- | --- | --- |
| Gender^a^ | 1.182* | .073 | < .001 | 3.260 (2.826 – 3.762) |
| Age | 0.075* | .033 | .0234 | 1.077 (1.010 – 1.149) |
| SES | -0.446* | .031 | < .0001 | 0.640 (0.602 – 0.680) |
| Sleep duration weekdays | -0.256* | .037 | < .0001 | 0.774 (0.720 – 0.833) |
| Sleep duration weekends | -0.007 | .038 | .8455 | 0.993 (0.920 – 1.070) |
| Sleep quality | -1.060* | .039 | < .0001 | 0.347 (0.321 – 0.374) |
| Chronotype | 0.104* | .038 | .0058 | 1.110 (1.031 – 1.195) |

*Note:* Sensitivity analysis was conducted for the adjusted regression model. b = beta coefficient. SE = standard error. OR = odds ratio. CI = confidence interval.

^a^Gender=girls (compared to boys)

*Statistically significant at p<.01

**S7D Table. Regression results when including outliers on bedtime and wake time variables.**

| **Variable** | **b** | **SE** | **p value** | **OR (95% CI)** |
| --- | --- | --- | --- | --- |
| Gender^a^ | 1.122* | 0.071 | < .0001 | 3.070 (2.674 – 3.530) |
| Age | 0.073* | 0.032 | .0247 | 1.076 (1.009 – 1.146) |
| SES | -0.449* | 0.030 | < .0001 | 0.638 (0.601 – 0.677) |
| Sleep duration weekdays | -0.327* | 0.043 | < .0001 | 0.721 (0.662 – 0.784) |
| Sleep duration weekends | -0.007 | 0.038 | .8625 | 0.993 (0.922 – 1.070) |
| Sleep quality | -1.111* | 0.039 | < .0001 | 0.329 (0.305 – 0.355) |
| Chronotype | 0.119* | 0.037 | .0013 | 1.126 (1.048 – 1.211) |

*Note:* Sensitivity analysis was conducted for the adjusted regression model. b = beta coefficient. SE = standard error. OR = odds ratio. CI = confidence interval.

^a^Gender=girls (compared to boys)
