# Supplementary material for: Associations between sleep habits, quality, chronotype and depression in a large cross-sectional sample of Swedish adolescents
Source: PLoS One. 2023 Nov 2;18(11):e0293580. doi: 10.1371/journal.pone.0293580 (PMC10621812; doi:10.1371/journal.pone.0293580)
Supplement: S8 Table — (DOCX) [file pone.0293580.s008.docx]

**S8 Table. STROBE checklist**

|  | Item No. | Recommendation | Page  No. | Relevant text from manuscript |
| --- | --- | --- | --- | --- |
| **Title and abstract** | 1 | (*a*) Indicate the study’s design with a commonly used term in the title or the abstract | 1 | Associations between sleep habits, quality, chronotype and depression in a large cross-sectional sample of Swedish adolescents |
|  |  | (*b*) Provide in the abstract an informative and balanced summary of what was done and what was found | 2 | Abstract |
| Introduction | | | |  |
| Background/rationale | 2 | Explain the scientific background and rationale for the investigation being reported | 3-7 | Sleep problems are common during adolescence and are a serious public health concern. It is recommended that 13-18-year-olds sleep between 8-10 hours per night regularly. Short sleep duration has become an increasing problem in many parts of the world. Apart from short sleep duration, many adolescents experience poor sleep quality. Sleep and mental health problems are closely interconnected, for example in diagnostic contexts. Improving the understanding of the relationship between sleep and depression in adolescents could contribute to public health efforts and could inform the development of preventive interventions and clinical treatment for depression. |
| Objectives | 3 | State specific objectives, including any prespecified hypotheses | 7,8 | The primary aim of this study was to describe behavioral sleep habits and self-perceived quality of sleep in a large Swedish sample of adolescents aged 12 to 16 years. The secondary aim was to investigate the relationship between different sleep parameters and clinically relevant levels of depression in this population. |
| Methods | | | |  |
| Study design | 4 | Present key elements of study design early in the paper | 8,9 | This study analyzed cross-sectional baseline data from a representative sample of adolescents aged 12-16 years in Stockholm County, Sweden. |
| Setting | 5 | Describe the setting, locations, and relevant dates, including periods of recruitment, exposure, follow-up, and data collection | 8,9 | Information was collected from participants during a cluster-randomized controlled trial evaluating a universal, school-based mental health promotion program in *n*=116 elementary and junior high schools (baseline measurement conducted while students attended grade 7 or 8) in Stockholm County between 2016 and 2020. Data were collected at baseline and at 3-month and 12-month follow-up. Participant recruitment and baseline data collection took place between August 2016 and November 2018. The present study included baseline data from participants in the control and intervention group who were between 12 and 16 years old at the time of enrolment (*n*=10288). The main analysis and descriptive statistics presented in the manuscript are from a sample of *n*=8449 participants (complete cases). |
| Participants | 6 | (*a*) *Cohort study*—Give the eligibility criteria, and the sources and methods of selection of participants. Describe methods of follow-up  *Case-control study*—Give the eligibility criteria, and the sources and methods of case ascertainment and control selection. Give the rationale for the choice of cases and controls  *Cross-sectional study*—Give the eligibility criteria, and the sources and methods of selection of participants | 9 | The study included participants from a cluster RCT evaluating a universal, school-based mental health promotion program. Individuals were attending grade 7 or 8 in elementary and junior high schools in Stockholm County. |
|  |  | (*b*) *Cohort study*—For matched studies, give matching criteria and number of exposed and unexposed  *Case-control study*—For matched studies, give matching criteria and the number of controls per case |  | N/A |
| Variables | 7 | Clearly define all outcomes, exposures, predictors, potential confounders, and effect modifiers. Give diagnostic criteria, if applicable | 10-15 | Outcome: Depression, with BDI scores > 13 indicating the presence of clinical symptoms of depression.  Predictors: Bedtime, wake time, and time in bed before falling asleep (sleep onset latency), sleep onset time, sleep duration, time in bed, for both weekdays and weekends. Sleep quality indicates the perceived quality of sleep. Chronotype reflects an estimation of the natural sleep rhythm of an individual.  Control variables: Socio-demographic information (gender, age, socio-economic status). |
| Data sources/ measurement | 8* | For each variable of interest, give sources of data and details of methods of assessment (measurement). Describe comparability of assessment methods if there is more than one group | 10-15 | Self-reported sleep habits were measured using the Karolinska Sleep Questionnaire (KSQ). A sleep quality index was calculated as the average score of 7 KSQ items. Chronotype was measured as the midpoint of sleep on weekends (corrected for sleep compensation). Depression was measured using the Beck Depression Inventory-II (BDI-II), with BDI scores > 13 indicating the presence of clinical symptoms of depression. As this study investigates the relationship between sleep and depression, the items “Changes in sleep patterns” and “Tiredness or fatigue” were excluded from the calculation of the BDI-II scores. In addition, the item “Loss of sexual interest” was excluded, as this item has been suggested to be unnecessary or psychometrically inappropriate in some adolescent contexts. Socio-economic status was measured using a proxy variable used in similar studies that indicates self-perceived relative economic status. |
| Bias | 9 | Describe any efforts to address potential sources of bias | 11 | Regarding information bias: Regarding the reported bedtimes, a minority of responses ranged between 7:00 and 13:00 (weekdays: *n*=1054; weekends: *n*=715) and these were considered as data entry errors reflecting answers on a 12-hour scale instead of the actual 24-hour scale (thus, e.g., a bedtime of 7:00 was corrected to 19:00). |
| Study size | 10 | Explain how the study size was arrived at | 9 | This study included all study participants from both the control and intervention group who were between 12 and 16 years old at the time of study enrolment (n= 10288). The main analysis and descriptive statistics presented in the manuscript are from a sample of *n*=8449 participants (complete cases). |

| Quantitative variables | 11 | Explain how quantitative variables were handled in the analyses. If applicable, describe which groupings were chosen and why | 14 | | A dichotomous depression variable was calculated, with BDI scores > 13 indicating the presence of clinical symptoms of depression. |
| --- | --- | --- | --- | --- | --- |
| Statistical methods | 12 | (*a*) Describe all statistical methods, including those used to control for confounding | 15-17 | | Binary logistic regression analysis was performed to investigate the adjusted relationships between the selected sleep parameters (sleep duration on weekdays, sleep duration on weekends, sleep quality, and chronotype) and depression. The adjusted model encompassed the control variables sex, age, and self-perceived socio-economic status. Bivariate effects were obtained from univariate regression models. |
|  |  | (*b*) Describe any methods used to examine subgroups and interactions | 16 | | Sleep parameters are reported for the total sample and disaggregated by gender and status of depression. T-tests and chi-square tests were performed to assess differences between boys and girls and the depressed and non-depressed group. |
|  |  | (*c*) Explain how missing data were addressed | 14, 16 | | Regarding the calculation of the BDI-II score: The modified BDI-II score was calculated across the 18 remaining items, allowing for a maximum of 2 missing items that were imputed by the average score. For the regression analysis, listwise deletion was used, resulting in a sample size of n=8449. |
|  |  | (*d*) *Cohort study*—If applicable, explain how loss to follow-up was addressed  *Case-control study*—If applicable, explain how matching of cases and controls was addressed  *Cross-sectional study*—If applicable, describe analytical methods taking account of sampling strategy |  | | N/A |
|  |  | (*e*) Describe any sensitivity analyses | 16, 17 | | Sensitivity analyses were conducted to assess whether excluding the BDI-II items “change in sleep patterns”, “tiredness or fatigue”, “loss of sexual interest” from the BDI-II scores had an impact on the results. Sensitivity analyses were also conducted for the sleep quality index excluding the items “nightmares” and “premature awakenings”. To ensure that linearity and normality were reasonable model assumptions, we also ran sensitivity tests with cubic and quadratic terms for the sleep duration variable. Finally, we assessed whether excluding outliers on the bedtime and wake time variables affected the results. |
| Results | | | | | |
| Participants | 13* | (a) Report numbers of individuals at each stage of study—eg numbers potentially eligible, examined for eligibility, confirmed eligible, included in the study, completing follow-up, and analysed  (b) Give reasons for non-participation at each stage  (c) Consider use of a flow diagram | 9 | | Participants included in the study: n=10288 (all participants aged 12-16 who participated in the original RCT) Participants included in the logistic regression model: n=8449 (number of participants that were included for each predictor variable is indicated in Table 3). Results reported in the main text of the manuscript refer to the sample of complete cases. However, in the supporting information, descriptive statistics are also presented for the total sample (n=10288). |
| Descriptive data | 14* | (a) Give characteristics of study participants (eg demographic, clinical, social) and information on exposures and potential confounders | 9, 10, 17-20 | | The study sample (n=8449) included 50.79% (n=4291) girls and 49.21% (n=4158) boys. The average age in the total sample was 14 years (SD=8 months). Self-perceived socioeconomic status can be interpreted as relatively high, as most participants reported that they often have enough money to do the same things as their friends (range 1-5, M=4.51, SD=0.82).  The average bedtime in the sample changed from 22:26 (*SD*=0:56) on weekdays to 24:08 (*SD*=1:33) on weekends, and the sleep onset time from 22:55 (*SD*=1:07) to 24:35 (*SD*=1:42). On weekdays, 45.6% of adolescents slept less than the recommended number of at least 8 hours per night; and 53.0% slept 8-10 hours, which is in accordance with the recommendations (Table 2). Among adolescents with depression, only 31.1% meet the sleep recommendations on weekdays, while this percentage is 58.7% in adolescents without depression. Sleep quality in the total sample, measured as the average score across 7 items on a 1-6 scale, was on average high (*M*=4.86, *SD*=0.84), indicating that most adolescents experienced good sleep quality (Fig 3). Chronotype was 04:51 in boys and 04:32 in girls. Adolescents with depression had a later chronotype (*M*=05:03, *SD*=1:28) than adolescents without depression (Fig 4). |
|  |  | (b) Indicate number of participants with missing data for each variable of interest | 22 | | For the adjusted regression model, we used complete cases (n=8449). For the bivariate effects from the unadjusted regression models (see Table 3) we report the number of participants underlying each of the effects. |
|  |  | (c) *Cohort study*—Summarise follow-up time (eg, average and total amount) |  | | N/A |
| Outcome data | 15* | *Cohort study*—Report numbers of outcome events or summary measures over time |  | | N/A |
|  |  | *Case-control study—*Report numbers in each exposure category, or summary measures of exposure |  | | N/A |
|  |  | *Cross-sectional study—*Report numbers of outcome events or summary measures | | 17 | Clinical levels of depression were prevalent in a total of 20.6% of adolescents (Fig 1). |
| Main results | 16 | (*a*) Give unadjusted estimates and, if applicable, confounder-adjusted estimates and their precision (eg, 95% confidence interval). Make clear which confounders were adjusted for and why they were included | 22 | | See Table 3. Unadjusted estimates see “Bivariate effects”.  “Adjusted model”: Girls were more likely to have clinical levels of depression (BDI-II scores > 13) than boys (OR=3.151, p<.0001). A one SD-increase in age (equivalent to 0.7 years) was associated with an 8%-increase in the odds of depression (OR=1.078, p=.0238). Higher self-perceived socioeconomic status (one SD equivalent to 0.9 scale points on a 1-5 scale) was associated with lower odds of depression (OR=0.645, p<.0001). For every SD-increase in sleep duration (equivalent to 1 hour and 12 minutes), the odds of having depression decreased by 23% (OR=0.773, p<.0001). Therefore, sleeping 30 minutes more on weekdays was associated with about 10% lower odds of having depression. Sleep duration on weekends however was not associated with depression (p=.9586). With a one SD-increase in sleep quality (equivalent to 0.9 score points on a 1-6 scale), the odds of having depression decreased by 67% (OR=0.327, p<.0001). Every SD-increase in chronotype (equivalent to 1 hour and 21 minutes) was associated with a 13% increase in the odds of having depression (OR=1.126, p=.0017). |
|  |  | (*b*) Report category boundaries when continuous variables were categorized |  | | Depression (BDI-II) scores were categorised (> 13 indicating the presence of clinical symptoms of depression), see Method section above |
|  |  | (*c*) If relevant, consider translating estimates of relative risk into absolute risk for a meaningful time period |  | | N/A |

| Other analyses | 17 | Report other analyses done—eg analyses of subgroups and interactions, and sensitivity analyses | 22 | Sensitivity analyses showed that excluding the items on sleep and sexual interest from the BDI score did not significantly affect the results. For the sleep quality index, sensitivity analyses confirmed that excluding the items on premature awakening and nightmares did not significantly change the effect of sleep quality on depression. We did not find evidence for a non-linear, u-shaped relationship between sleep duration and depression, by including a quadratic and cubic term for sleep duration in the regression model. Finally, trimming the bedtime and wake time variables did not significantly affect the results either. |
| --- | --- | --- | --- | --- |
| Discussion | | | | |
| Key results | 18 | Summarise key results with reference to study objectives | 23-29 | In this study, the average sleep duration on weekdays (7:53h) was below the recommended 8 hours of sleep per night for this age group. These findings confirm that short sleep duration is a prevalent issue among 12-16-year-olds in Sweden and other countries around the world.  Notably, we found that this social schedule did not align with the adolescents’ chronotypes at all, as it would imply that adolescents need to wake up and start school with their inner biological clock still being set at night-time. Only about 7% of adolescents in this sample had an optimal chronotype that was compatible with school-start times that require waking up at 7 a.m. and that allowed them to receive at least 8 hours of sleep. Girls experienced issues such as difficulties falling asleep and waking up, and not feeling well-rested on awakenings more frequently than boys.  Results from our regression analysis in this study confirmed findings of previous studies showing that there is a negative association between sleep duration on weekdays and depression in adolescents. Sleep quality was the strongest sleep-related predictor of depression in our regression model. Late chronotype was associated with depression, even after adjusting for sleep duration and sleep quality. |
| Limitations | 19 | Discuss limitations of the study, taking into account sources of potential bias or imprecision. Discuss both direction and magnitude of any potential bias | 30, 31 | The primary weakness of this study is its cross-sectional design, which makes it difficult to draw conclusions about causality. Further studies using longitudinal designs with moderation and mediation analyses could clarify the causal pathways between sleep parameters and depression. Another limitation of this study is the use of self-report measures of sleep behaviors, which appear to overestimate sleep duration compared to actigraphy and polysomnography. Self-report measures may, however, be more appropriate to measure sleep quality, which reflects subjectively perceived quality of sleep rather than objective sleep behavior (which we actually found to be a better predictor for depression than duration). |
| Interpretation | 20 | Give a cautious overall interpretation of results considering objectives, limitations, multiplicity of analyses, results from similar studies, and other relevant evidence | 28, 29 | Although the causal mechanisms are not yet fully understood, it seems likely that sleep plays a critical role in the development of depression. It is important to identify factors that obstruct healthy sleep habits, and methods that promote sleep duration and sleep quality in adolescents. Determinants may include social factors like early school start times, which may play a central role, especially for individuals that have a late chronotype. In fact, delaying school start times has been shown to increase adolescents’ sleep duration and improve academic performance. Additional behavioral factors may of course also play an important role in the relationship between sleep and depression, for example screen time. |
| Generalisability | 21 | Discuss the generalisability (external validity) of the study results | 30 | The sampling strategy enrolled schools from different socioeconomic areas in Stockholm County, which contributes to the generalizability of the findings presented in this study. |
| Other information | |  | | |
| Funding | 22 | Give the source of funding and the role of the funders for the present study and, if applicable, for the original study on which the present article is based |  | The execution of this study was not funded by any research grants. The salaries of the  authors were paid within the frame of their standard employments. |

*Give information separately for cases and controls in case-control studies and, if applicable, for exposed and unexposed groups in cohort and cross-sectional studies.

**Note:** An Explanation and Elaboration article discusses each checklist item and gives methodological background and published examples of transparent reporting. The STROBE checklist is best used in conjunction with this article (freely available on the Web sites of PLoS Medicine at http://www.plosmedicine.org/, Annals of Internal Medicine at http://www.annals.org/, and Epidemiology at http://www.epidem.com/). Information on the STROBE Initiative is available at www.strobe-statement.org.
