# Supplementary material for: Associations between sleep habits, quality, chronotype and depression in a large cross-sectional sample of Swedish adolescents
Source: PLoS One. 2023 Nov 2;18(11):e0293580. doi: 10.1371/journal.pone.0293580 (PMC10621812; doi:10.1371/journal.pone.0293580)
Supplement: S9 Table — N = 10288 (total sample of participants with baseline data, aged 12–16 years old). Depression: BDI-II scores as a continuous variable. aweekdays. *Correlation is significant at the 0.01 level. (DOCX) [file pone.0293580.s009.docx]

**S9 Table. Bivariate Pearson correlations for weekday sleep variables in the total sample.**

|  | Depression | Bedtime^a^ | Sleep onset latency^a^ | Sleep onset time^a^ | Wake time^a^ | Sleep duration^a^ | Time in bed^a^ | Chronotype |
| --- | --- | --- | --- | --- | --- | --- | --- | --- |
| Bedtime^a^ | .218* | - |  |  |  |  |  |  |
| Sleep onset latency^a^ | .304* | .097* | - |  |  |  |  |  |
| Sleep onset time^a^ | .319* | .879* | .559* | - |  |  |  |  |
| Wake time^a^ | -.163* | .128* | -.049* | .087* | - |  |  |  |
| Sleep duration^a^ | -.372* | -.764* | -.545* | -.897* | .362* | - |  |  |
| Time in bed^a^ | -.290* | -.853* | -.116* | -.766* | .408* | .896* | - |  |
| Chronotype | .174* | .440* | .231* | .477* | .190* | -.363* | -.308* | - |
| Sleep quality | -.636* | -.245* | -.368* | -.372* | .097* | .396* | .282* | -.190* |

*Note:* N=10288 (total sample of participants with baseline data, aged 12-16 years old).
Depression: BDI-II scores as a continuous variable.
^a^weekdays

*Correlation is significant at the 0.01 level.
