# Supplementary material for: Associations between sleep habits, quality, chronotype and depression in a large cross-sectional sample of Swedish adolescents
Source: PLoS One. 2023 Nov 2;18(11):e0293580. doi: 10.1371/journal.pone.0293580 (PMC10621812; doi:10.1371/journal.pone.0293580)
Supplement: S10 Table — N = 10288 (total sample of participants with baseline data, aged 12–16 years old). Depression: BDI-II scores as a continuous variable. aweekends. *Correlation is significant at the 0.01 level. (DOCX) [file pone.0293580.s010.docx]

**S10 Table. Bivariate Pearson correlations for weekend sleep variables in the total sample.**

|  | Depression | Bedtime^a^ | Sleep onset latency^a^ | Sleep onset time^a^ | Wake time^a^ | Sleep duration^a^ | Time in bed^a^ | Chronotype |
| --- | --- | --- | --- | --- | --- | --- | --- | --- |
| Bedtime^a^ | .219* | - |  |  |  |  |  |  |
| Sleep onset latency^a^ | .214* | .113* | - |  |  |  |  |  |
| Sleep onset time^a^ | .270* | .939* | .447* | - |  |  |  |  |
| Wake time^a^ | .119* | .492* | .117* | .492* | - |  |  |  |
| Sleep duration^a^ | -.187* | -.570* | -.387* | -.647* | .346* | - |  |  |
| Time in bed^a^ | -.118* | -.576* | -.017 | -.521** | .428* | .929* | - |  |
| Chronotype | .174* | .917* | .347* | .950* | .651* | -.445* | -.338* | - |
| Sleep quality | -.636* | -.228* | -.250* | -.287* | -.134* | .199* | .115* | -.190* |

*Note:* N=10288 (total sample of participants with baseline data, aged 12-16 years old).
Depression: BDI-II scores as a continuous variable.
^a^weekends

*Correlation is significant at the 0.01 level.
