# Supplementary material for: Associations between sleep habits, quality, chronotype and depression in a large cross-sectional sample of Swedish adolescents
Source: PLoS One. 2023 Nov 2;18(11):e0293580. doi: 10.1371/journal.pone.0293580 (PMC10621812; doi:10.1371/journal.pone.0293580)
Supplement: S11 Table — Total sample = Participants with baseline data, aged 12–16 years old. Boys and girls are compared using t-tests. M = Mean. Mdn = Median. SD = Standard deviation. SE (M) = standard error of the mean. Sleep habits and duration in hh:mm format. Sleep quality: Average sleep quality index score, range 1–6 (higher scores indicate better sleep quality). P-value based on t-test, comparing boys and girls. a t-test for unequal variances. *p < 0.05. (DOCX) [file pone.0293580.s011.docx]

**S11 Table. Sleep habits and sleep quality in girls and boys in the total sample (n=10288), M (SD).**

|  | **Total** (N = 10288) | **SE (M)** | **Boys** (N = 5179) | **Girls** (N = 5020) | **p value** | **Mean difference (SE)** |
| --- | --- | --- | --- | --- | --- | --- |
| **Weekdays** |  |  |  |  |  |  |
| Bedtime | 22:27 (0:57) | 0:00 | 22:25 (0:57) | 22:29 (0:56) | p = .0008, t = -3.367 | -0:03 (0:01) |
| Sleep onset latency | 00:30 (0:34) | 0:00 | 00:28 (0:33) | 00:32 (0:34) | p < .0001^a^, t = -6.214 | -0:04 (0:01) |
| Sleep onset time | 22:56 (1:08) | 0:00 | 22:52 (1:07) | 23:00 (1:08) | p < .0001, t = -5.527 | -0:07 (0:01) |
| Wake time | 6:48 (0:32) | 0:00 | 6:56 (0:31) | 6:40 (0:31) | p < .0001^a^, t = 25.286 | 0:16 (0:00) |
| Sleep duration | 7:52 (1:12) | 0:00 | 8:04 (1:09) | 7:40 (1:14) | p < .0001^a^, t = 16.185 | 0:24 (0:01) |
| Time in bed | 8:21 (1:01) | 0:00 | 8:31 (0:59) | 8:11 (1:02) | p < .0001^a^, t = 16.327 | 0:20 (0:01) |
|  |  |  |  |  |  |  |
| **Weekends** |  |  |  |  |  |  |
| Bedtime | 24:09 (1:37) | 0:00 | 24:14 (1:40) | 24:04 (1:33) | p < .0001^a^, t = 5.298 | 0:10 (0:01) |
| Sleep onset latency | 00:29 (0:38) | 0:00 | 00:28 (0:36) | 00:31 (0:39) | p = .0002^a^, t = -3.739 | -0:03 (0:01) |
| Sleep onset time | 24:38 (1:47) | 0:01 | 24:43 (1:49) | 24:33 (1:45) | p = .0001^a^, t = 4.138 | 0:09 (0:02) |
| Wake time | 9:57 (1:28) | 0:00 | 10:04 (1:30) | 9:49 (1:24) | p < .0001^a^, t = 8.775 | 0:15 (0:01) |
| Sleep duration | 9:17 (1:39) | 0:01 | 9:20 (1:39) | 9:14 (1:37) | p = .0026, t = 3.008 | 0:06 (0:02) |
| Time in bed | 9:47 (1:33) | 0:00 | 9:49 (1:35) | 9:44 (1:30) | p = .0162^a^, t = 2.405 | 0:04 (0:01) |
|  |  |  |  |  |  |  |
| **Chronotype** | 04:42 (1:21) | 0:00 | 04:51 (1:23) | 04:33 (1:17) | p < .0001^a^, t = 10.627 | 0:18 (0:01) |
| **Sleep quality** | 4.84 (0.88) | .009 | 5.06 (0.76) | 4.63 (0.92) | p < .0001^a^, t = 24.932 | 0.43 (.017) |
| **Single items of the sleep quality index** | | | |  |  |  |
| 1. Difficulties  falling asleep | 4.55 (1.41) Mdn: 5.00 | .014 | 4.84 (1.26) Mdn: 5.00 | 4.27 (1.48) Mdn: 5.00 | p < .0001^a^, t = 20.609 | 0.57 (.028) |
| 2. Difficulties  waking up | 4.10 (1.68) Mdn: 5.00 | .017 | 4.37 (1.62) Mdn: 5.00 | 3.85 (1.70) Mdn: 4.00 | p < .0001^a^, t = 15.441 | 0.52 (.034) |
| 3. Repeated awakenings with difficulties falling asleep again | 5.19 (1.16) Mdn: 6.00 | .012 | 5.35 (1.04) Mdn: 6.00 | 5.03 (1.24) Mdn: 5.00 | p < .0001^a^, t = 13.607 | 0.32 (.023) |
| 4. Nightmares | 5.37 (1.00) Mdn: 6.00 | .010 | 5.56 (0.84)  Mdn: 6.00 | 5.19 (1.10) Mdn: 6.00 | p < .0001^a^, t = 18.542 | 0.37 (.020) |
| 5. Not well-rested on awakenings | 4.27 (1.61) Mdn: 5.00 | .016 | 4.58 (1.48)  Mdn: 5.00 | 3.97 (1.67) Mdn: 4.00 | p < .0001^a^, t = 18.929 | 0.61 (.032) |
| 6. Premature awakenings | 5.00 (1.19) Mdn: 5.00 | .012 | 5.12 (1.11) Mdn: 5.00 | 4.90 (1.25) Mdn: 5.00 | p < .0001^a^, t = 9.187 | 0.22 (.024) |
| 7. Disturbed/ restless sleep | 5.36 (1.08)  Mdn: 6.00 | .011 | 5.55 (0.89)  Mdn: 6.00 | 5.17 (1.20) Mdn: 6.00 | p < .0001^a^, t = 18.030 | 0.39 (.022) |

*Note:* Total sample = Participants with baseline data, aged 12-16 years old.
Boys and girls are compared using t-tests. M = Mean. Mdn = Median. SD = Standard deviation. SE (M) = standard error of the mean. Sleep habits and duration in hh:mm format.
Sleep quality: Average sleep quality index score, range 1-6 (higher scores indicate better sleep quality).
P-value based on t-test, comparing boys and girls.

^a^ t-test for unequal variances.

*p < 0.05.
