# Supplementary material for: Associations between sleep habits, quality, chronotype and depression in a large cross-sectional sample of Swedish adolescents
Source: PLoS One. 2023 Nov 2;18(11):e0293580. doi: 10.1371/journal.pone.0293580 (PMC10621812; doi:10.1371/journal.pone.0293580)
Supplement: S12 Table — Total sample = Participants with baseline data, aged 12–16 years old. Depressed and non-depressed groups are compared using t-tests. M = Mean. SD = Standard deviation. Mdn = Median. Sleep habits and duration in hh:mm format. Sleep quality: Average sleep quality index score, range 1–6 (higher scores indicate better sleep quality). BDI-II: modified Beck Depression Inventory-II (excluding three items), range 0–63. P-value based on t-test, comparing depressed and non-depressed. a t-test for unequal variances. (DOCX) [file pone.0293580.s012.docx]

**S12 Table. Sleep habits and sleep quality in non-depressed (BDI-II ≤ 13) and depressed (BDI-II > 13) adolescents in the total sample (n=10288), by gender, M (SD).**

|  | **Total** (N = 10288) | | |  | **Boys** (N = 5179) | | |  | **Girls** (N = 5020) | | |
| --- | --- | --- | --- | --- | --- | --- | --- | --- | --- | --- | --- |
|  | **BDI**  **≤ 13** | **BDI  > 13** | **P value** |  | **BDI  ≤ 13** | **BDI  > 13** | **P value** |  | **BDI  ≤ 13** | **BDI  > 13** | **P value** |
| **Weekdays** | | | | | | | | | | | |
| Bedtime | 22:22  (0:53) | 22:46 (1:04) | p < .0001^a^, t = -15.437 |  | 22:23 (0:55) | 22:43  (1:06) | p < .0001^a^, t = -6.522 |  | 22:20  (0:50) | 22:47  (1:04) | p < .0001^a^, t = -14.203 |
| Sleep onset latency | 0:25 (0:28) | 0:46 (0:45) | p < .0001^a^, t = -19.484 |  | 0:25 (0:29) | 0:47  (0:46) | p < .0001^a^, t = -10.238 |  | 0:26  (0:26) | 0:45  (0:44) | p < .0001^a^, t = -15.783 |
| Sleep onset time | 22:47  (1:02) | 23:30  (1:17) | p < .0001^a^, t = -22.525 |  | 22:47  (1:04) | 23:29  (1:21) | p < .0001^a^, t = -10.902 |  | 22:46  (0:59) | 23:30  (1:17) | p < .0001^a^, t = -19.223 |
| Wake time | 6:50  (0:30) | 6:40  (0:36) | p < .0001^a^, t = 12.088 |  | 6:57  (0:30) | 6:52  (0:36) | p < .0139^a^, t = 2.466 |  | 6:42  (0:28) | 6:36  (0:36) | p < .0001^a^, t = 6.139 |
| Sleep duration | 8:03 (1:04) | 7:10  (1:24) | p < .0001^a^, t = 26.092 |  | 8:09 (1:05) | 7:23  (1:25) | p < .0001^a^, t = 11.239 |  | 7:56  (1:03) | 7:06  (1:23) | p < .0001^a^, t = 20.444 |
| Time in bed | 8:28 (0:56) | 7:53  (1:11) | p < .0001^a^, t = 20.345 |  | 8:34  (0:57) | 8:09  (1:09) | p < .0001^a^, t = 7.527 |  | 8:21  (0:55) | 7:48  (1:11) | p < .0001^a^, t = 15.926 |
| **Weekends** | | | | | | | | | | | |
| Bedtime | 00:01 (1:32) | 00:40  (1:48) | p < .0001^a^, t = -15.158 |  | 00:10 (1:37) | 00:50  (1:55) | p < .0001^a^, t = -7.407 |  | 23:48  (1:22) | 00:37  (1:45) | p < .0001^a^, t = -15.846 |
| Sleep onset latency | 0:26 (0:33) | 0:41 (0:48) | p < .0001^a^, t = -13.586 |  | 0:26 (0:34) | 0:40  (0:48) | p < .0001^a^, t = -6.323 |  | 0:25 (0:32) | 0:41  (0:48) | p < .0001^a^, t = -11.504 |
| Sleep onset time | 00:26 (1:40) | 01:21  (2:01) | p < .0001^a^, t = -18.337 |  | 00:36  (1:44) | 01:34 (2:03) | p < .0001^a^, t = -9.590 |  | 00:13  (1:31) | 01:16  (2:00) | p < .0001^a^, t = -17.740 |
| Wake time | 9:52 (1:24) | 10:12  (1:38) | p < .0001^a^, t = -8.316 |  | 10:01  (1:28) | 10:26  (1:43) | p < .0001^a^, t = -5.040 |  | 9:39  (1:15) | 10:08  (1:36) | p < .0001^a^, t = -10.061 |
| Sleep duration | 9:24 (1:31) | 8:49  (1:58) | p < .0001^a^, t = 12.184 |  | 9:23  (1:35) | 8:50  (2:02) | p < .0001^a^, t = 5.579 |  | 9:25  (1:26) | 8:50  (1:55) | p < .0001^a^, t = 10.351 |
| Time in bed | 9:51  (1:26) | 9:31  (1:49) | p < .0001^a^, t = 7.556 |  | 9:50  (1:31) | 9:35  (1:54) | p < .0001^a^, t = 2.880 |  | 9:51  (1:20) | 9:30  (1:47) | p < .0001^a^, t = 6.646 |
| **Chronotype** | 04:36 (1:17) | 05:02  (1:28) | p < .0001^a^, t = -11.532 |  | 04:48  (1:21) | 05:19  (1:31) | p < .0001^a^, t = -6.735 |  | 04:22  (1:10) | 04:57  (1:27) | p < .0001^a^, t = -13.197 |
|  |  |  |  |  |  |  |  |  |  |  |  |
| **Sleep quality** | 5.07  (0.68) | 3.99  (0.97) | p < .0001^a^, t = 47.422 |  | 5.17  (0.65) | 4.18 (0.94) | p < .0001^a^, t = 22.554 |  | 4.95 (0.69) | 3.94 (0.96) | p < .0001^a^, t = 36.724 |
| **Single items of the sleep quality index** | | | |  |  |  |  |  |  |  |  |
| 1. Difficulties  falling asleep | 4.84 (1.18) Mdn: 5 | 3.52  (1.63)  Mdn: 4 | p < .0001^a^, t = 34.649 |  | 4.97 (1.13) Mdn: 5 | 3.77 (1.65)  Mdn: 4 | p < .0001^a^, t = 16.023 |  | 4.66 (1.23)  Mdn: 5 | 3.45 (1.61)  Mdn: 4 | p < .0001^a^, t = 26.241 |
| 2. Difficulties  waking up | 4.37  (1.57) Mdn: 5 | 3.14  (1.71)  Mdn: 3 | p < .0001^a^, t = 29.757 |  | 4.50 (1.56) Mdn: 5 | 3.29 (1.73)  Mdn: 3 | p < .0001^a^, t = 15.120 |  | 4.21 (1.57)  Mdn: 5 | 3.10 (1.70)  Mdn: 3 | p < .0001^a^, t = 21.489 |
| 3. Repeated awakenings with difficulties falling asleep again | 5.41  (0.92)  Mdn: 6 | 4.42  (1.51)  Mdn: 5 | p < .0001^a^, t = 28.571 |  | 5.46 (0.90)  Mdn: 6 | 4.49 (1.52)  Mdn: 5 | p < .0001^a^, t = 14.016 |  | 5.34 (0.94)  Mdn: 6 | 4.40 (1.51)  Mdn: 5 | p < .0001^a^, t = 22.541 |
| 4. Nightmares | 5.54  (0.77)  Mdn: 6 | 4.76  (1.38)  Mdn: 5 | p < .0001^a^, t = 24.835 |  | 5.63  (0.71)  Mdn: 6 | 4.97 (1.34)  Mdn: 5 | p < .0001^a^, t = 10.988 |  | 5.43 (0.83)  Mdn: 6 | 4.70 (1.38) Mdn: 5 | p < .0001^a^, t = 19.075 |
| 5. Not well-rested on awakenings | 4.59  (1.43)  Mdn: 5 | 3.13  (1.70)  Mdn: 3 | p < .0001^a^, t = 35.777 |  | 4.72  (1.39)  Mdn: 5 | 3.47 (1.69)  Mdn: 4 | p < .0001^a^, t = 15.998 |  | 4.42 (1.46)  Mdn: 5 | 3.03 (1.69) Mdn: 3 | p < .0001^a^, t = 27.738 |
| 6. Premature awakenings | 5.16  (1.03)  Mdn: 5 | 4.45  (1.50)  Mdn: 5 | p < .0001^a^, t = 20.483 |  | 5.19 (1.03) Mdn: 5 | 4.51  (1.47)  Mdn: 5 | p < .0001^a^, t = 10.052 |  | 5.12 (1.03)  Mdn: 5 | 4.44 (1.51) Mdn: 5 | p < .0001^a^, t = 16.059 |
| 7. Disturbed/ restless sleep | 5.59  (0.78)  Mdn: 6 | 4.52  (1.50)  Mdn: 5 | p < .0001^a^, t = 31.221 |  | 5.66  (0.73)  Mdn: 6 | 4.74  (1.44)  Mdn: 5 | p < .0001^a^, t = 14.133 |  | 5.50 (0.83)  Mdn: 6 | 4.47 (1.51) Mdn: 5 | p < .0001^a^, t = 25.075 |

*Note:* Total sample = Participants with baseline data, aged 12-16 years old.
Depressed and non-depressed group are compared using t-tests.

M = Mean. SD = Standard deviation. Mdn = Median.

Sleep habits and duration in hh:mm format.

Sleep quality: Average sleep quality index score, range 1-6 (higher scores indicate better sleep quality).

BDI-II: modified Beck Depression Inventory-II (excluding three items), range 0-63.

P-value based on t-test, comparing depressed and non-depressed.

^a^ t-test for unequal variances.
